# A Trigger Enzyme in *Mycoplasma pneumoniae*: Impact of the Glycerophosphodiesterase GlpQ on Virulence and Gene Expression

**DOI:** 10.1371/journal.ppat.1002263

**Published:** 2011-09-22

**Authors:** Sebastian R. Schmidl, Andreas Otto, Maria Lluch-Senar, Jaume Piñol, Julia Busse, Dörte Becher, Jörg Stülke

**Affiliations:** 1 Department of General Microbiology, Georg-August-University Göttingen, Göttingen, Germany; 2 Institut für Mikrobiologie und Molekularbiologie, Ernst-Moritz-Arndt-Universität Greifswald, Greifswald, Germany; 3 Institut de Biotecnologia i Biomedicina and Departament de Bioquímica i Biologia Molecular, Universitat Autònoma de Barcelona, Barcelona, Spain; University of Pittsburgh School of Medicine, United States of America

## Abstract

*Mycoplasma pneumoniae* is a causative agent of atypical pneumonia. The formation of hydrogen peroxide, a product of glycerol metabolism, is essential for host cell cytotoxicity. Phosphatidylcholine is the major carbon source available on lung epithelia, and its utilization requires the cleavage of deacylated phospholipids to glycerol-3-phosphate and choline. *M. pneumoniae* possesses two potential glycerophosphodiesterases, MPN420 (GlpQ) and MPN566. In this work, the function of these proteins was analyzed by biochemical, genetic, and physiological studies. The results indicate that only GlpQ is an active glycerophosphodiesterase. MPN566 has no enzymatic activity as glycerophosphodiesterase and the inactivation of the gene did not result in any detectable phenotype. Inactivation of the *glpQ* gene resulted in reduced growth in medium with glucose as the carbon source, in loss of hydrogen peroxide production when phosphatidylcholine was present, and in a complete loss of cytotoxicity towards HeLa cells. All these phenotypes were reverted upon complementation of the mutant. Moreover, the *glpQ* mutant strain exhibited a reduced gliding velocity. A comparison of the proteomes of the wild type strain and the *glpQ* mutant revealed that this enzyme is also implicated in the control of gene expression. Several proteins were present in higher or lower amounts in the mutant. This apparent regulation by GlpQ is exerted at the level of transcription as determined by mRNA slot blot analyses. All genes subject to GlpQ-dependent control have a conserved potential *cis*-acting element upstream of the coding region. This element overlaps the promoter in the case of the genes that are repressed in a GlpQ-dependent manner and it is located upstream of the promoter for GlpQ-activated genes. We may suggest that GlpQ acts as a trigger enzyme that measures the availability of its product glycerol-3-phosphate and uses this information to differentially control gene expression.

## Author Summary

*Mycoplasma pneumoniae* serves as a model organism for bacteria with very small genomes that are nonetheless independently viable. These bacteria infect the human lung and cause an atypical pneumonia. The major virulence determinant of *M. pneumoniae* is hydrogen peroxide that is generated during the utilization of glycerol-3-phosphate, which might be derived from free glycerol or from the degradation of phospholipids. Indeed, lecithin is the by far most abundant carbon source on lung epithelia. In this study, we made use of the recent availability of methods to isolate mutants of *M. pneumoniae* and characterized the enzyme that generates glycerol-3-phosphate from deacylated lecithin (glycerophosphocholine). This enzyme, called GlpQ, is essential for the formation of hydrogen peroxide when the bacteria are incubated with glycerophosphocholine. Moreover, *M. pneumoniae* is unable to cause any detectable damage to the host cells in the absence of GlpQ. This underlines the important role of phospholipid metabolism for the virulence of *M. pneumoniae*. We observed that GlpQ in addition to its enzymatic activity is also involved in the control of expression of several genes, among them the glycerol transporter. Thus, GlpQ is central to the normal physiology and to pathogenicity of the minimal pathogen *M. pneumoniae*.

## Introduction

Pathogenic bacteria have developed a large battery of enzymes and mechanisms for extracting nutrients from their hosts, and the requirement for nutrient acquisition can be regarded as one of the driving forces for virulence [1]–[3]. In consequence, the metabolic capabilities of a pathogen reflect its adaptation to a particular niche in a particular host.

*Mycoplasma pneumoniae* is a causative agent of atypical pneumonia, however implication of this bacterium in several additional infections including encephalitis, aseptic meningitis, acute transverses myelitis, stroke, and polyradiculopathy has been reported [4]–[7]. These bacteria are members of the taxonomic class *Mollicutes* that are characterized by an extreme reductive evolution that results in the smallest genomes that allow independent life. Moreover, the mollicutes have lost the cell wall and most metabolic pathways, since they obtain the building blocks for their cellular macromolecules from the host tissue. However, even in these minimal pathogens, there is a close relation between metabolism and virulence (for a review, see [8]). *M. pneumoniae* thrives at the apical surface of lung epithelia. Thus, these bacteria must have evolved to utilize the carbon sources present in this niche. The pulmonary surfactant is composed of about 90% phospholipids and 10% proteins [9]. This suggests that phospholipids play a major role in the nutrition of *M. pneumoniae*.

**Figure 1:**
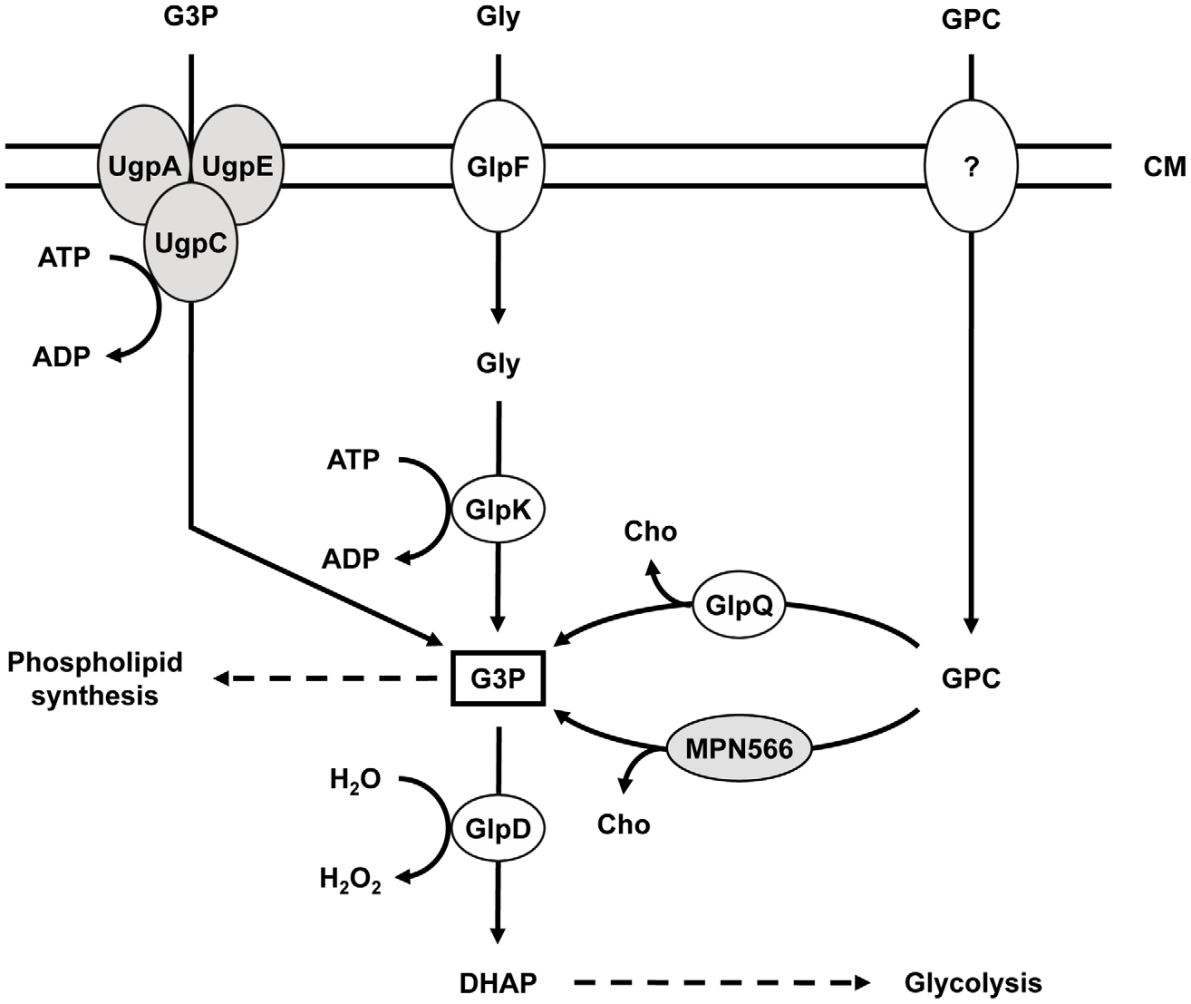
Schematic illustration of the machinery for uptake and conversion of carbohydrates leading to the formation of glycerol-3-phosphate in *M. pneumoniae*. UgpC (MPN134), UgpA (MPN135), and UgpE (MPN136) form a putative ABC transport system for glycerol-3-phosphate, whereas GlpF (MPN043) is the glycerol uptake facilitator. The glycerol kinase GlpK (MPN050) and the glycerol-3-phosphate oxidase GlpD (MPN051) metabolize glycerol to the glycolytic intermediate dihydroxyacetone phosphate. Hydrogen peroxide formation by GlpD is crucial for the cytotoxic effects of *M. pneumoniae*. GlpQ (MPN420) and MPN566 encode two paralogous glycerophosphodiesterases that may be able to metabolize glycerophosphocholine to glycerol-3-phosphate and choline. The uptake system for glycerophosphocholine is so far unknown. Proteins highlighted in grey seem not to fulfill the predicted function (this work). CM, cell membrane; DHAP, dihydroxyacetone phosphate; G3P, glycerol-3-phosphate; GPC, glycerophosphocholine; Gly, glycerol; Cho, choline.

Glycerophospholipids, the major building blocks of the cell membrane in bacteria and eukaryotes, are degraded in several steps. First, the fatty acids are cleaved from the phospholipids resulting in the formation of glycerophosphodiesters. In these molecules, the phosphate group of glycerol-3-phosphate is linked to another compound, called the head group. In eukaryotes, choline is by far the most abundant head group, and lecithin, the choline-containing phospholipid accounts for about 80% of all phospholipids in human lung cells [9]. In the second step, the choline head group is cleaved due to the activity of a glycerophosphodiesterase resulting in the formation of glycerol-3-phosphate that can feed into glycolysis after oxidation to dihydroxyacetone phosphate (see Figure **1**). In *M. pneumoniae*, the latter reaction is catalyzed by the glycerol-3-phosphate oxidase GlpD [10]. GlpD transfers the electrons to water resulting in the formation of hydrogen peroxide, the major virulence factor of *M. pneumoniae* [11]. In consequence, the virulence of *M. pneumoniae glpD* mutant cells is severely attenuated [10].

While the metabolism of glycerol has been well studied in *M. pneumoniae* and other mollicutes such as *M. mycoides* [10], [12], [13], only little is known about the glycerophosphodiesterases required for lipid utilization. Many bacteria encode multiple glycerophosphodiesterases. In *E. coli*, both enzymes are enzymatically active in lipid degradation; however, they are differentially regulated, with GlpQ and UgpQ being induced in the presence of glycerol-3-phosphate and under conditions of phosphate starvation, respectively [14], [15]. In *B. subtilis*, out of three putative glycerophosphodiesterases, only GlpQ has been studied. The corresponding gene is under dual control and its expression is induced when phosphate becomes limiting and glycerol is available [16], [17]. Moreover, *glpQ* expression is repressed if more favourable carbon sources such as glucose are present [18]. In *Haemophilus influenzae*, another bacterium thriving in the respiratory tract, the glycerophosphodiesterase is involved in pathogenicity. The enzyme generates choline, which in turn is used for the biosynthesis of the bacterial lipopolysaccharide layer - a major virulence determinant of Gram-negative bacteria [19], [20]. Similarly, glycerophosphodiesterase activity is implicated in virulence of different *Borrelia* species. The enzyme is only present in the relapsing fever group and may help the bacteria to reach a higher cell density in the blood of the host as compared to Lyme disease spirochaetes [21].

In many bacteria, central enzymes of metabolism do not only fulfil their catalytic function, but in addition, they are also involved in signal transduction. In this way, the information on the availability of important metabolites can be directly determined by the enzyme in charge of their conversion, and this information is then often transferred to the transcription machinery. Collectively, such enzymes have been termed trigger enzymes [22]. They can control gene expression by directly acting as DNA- or RNA-binding transcription factors as the *E. coli* proline dehydrogenase and the aconitase or by controlling the activity of transcription factors by covalent modification or a regulatory protein-protein interaction as observed for several sugar permeases of the bacterial phosphotransferase system and the *B. subtilis* glutamate dehydrogenase, respectively [23]–[26].

In this work, we have analyzed the role of the two potential glycerophosphodiesterases encoded in the genome of *M. pneumoniae*. Biochemical and physiological studies demonstrate that one of the two proteins, GlpQ, is a functional glycerophosphodiesterase. GlpQ is essential for hydrogen peroxide formation in the presence of deacylated phospholipids as the carbon source and, in consequence, for cytotoxicity. Moreover, GlpQ may act as a trigger enzyme by controlling the expression of a set of genes encoding lipoproteins, the glycerol facilitator, and a metal ion ABC transporter.

## Results

### Identification of *M. pneumoniae* genes encoding potential glycerophosphodiesterases

Since phospholipids are the most abundant potential carbon sources for *M. pneumoniae* living at lung epithelial surfaces, we considered the possibility that these bacteria synthesize enzymes that cleave the polar head groups from the glycerophosphodiesters to produce glycerol-3-phosphate that can be utilized by the enzymes of glycerol metabolism [10] (Figure **1**). Two genes that potentially encode such enzymes are present in the genome of *M. pneumoniae*, *i.e. mpn420* (renamed to *glpQ*) and *mpn566*. An alignment of the corresponding proteins to glycerophosphodiesterases from other bacteria is shown in the supporting information (Figure **S1**).

### Enzymatic activities of the potential glycerophosphodiesterases

**Figure 2:**
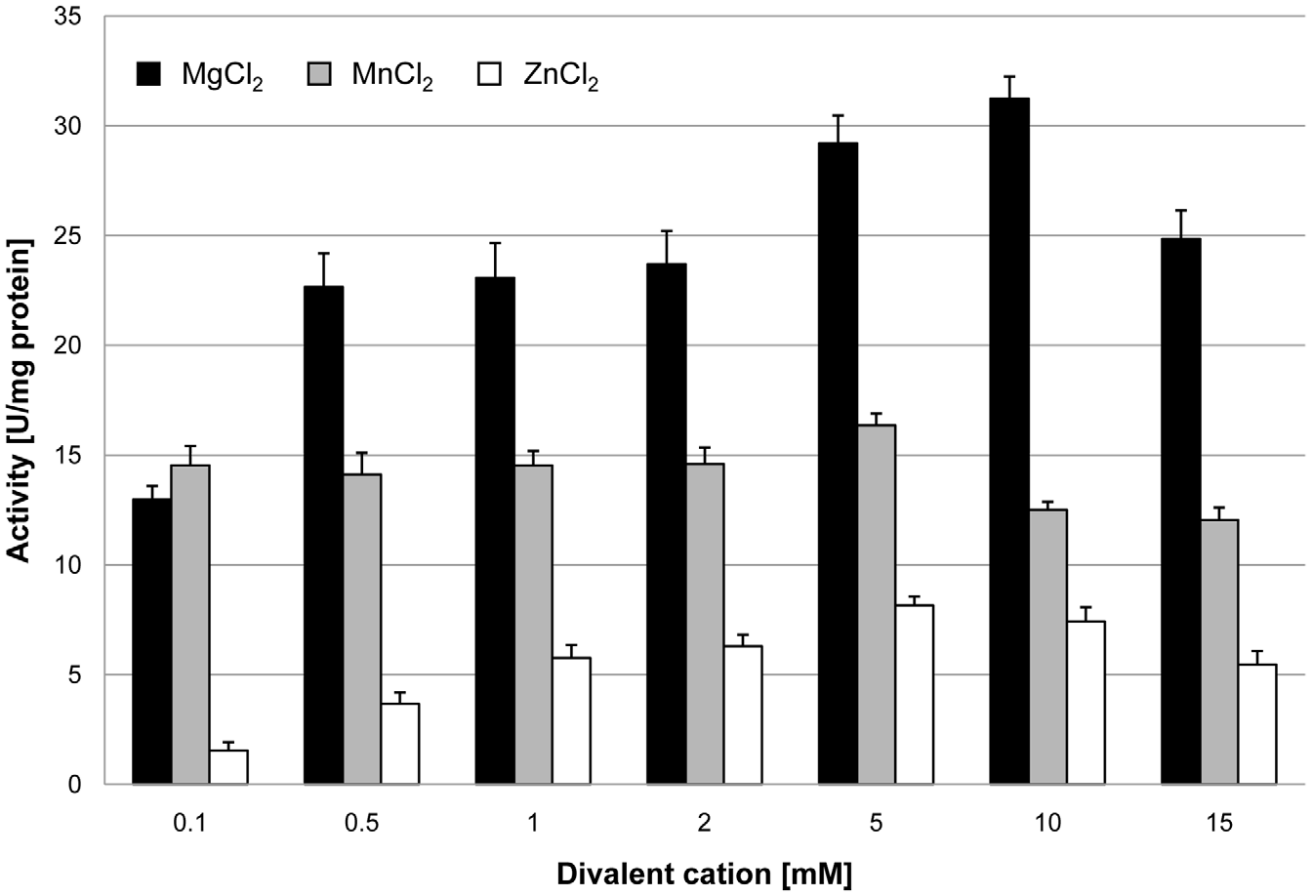
Effect of divalent cations on GlpQ activity. GlpQ activity was measured in the presence of 0.5 mM glycerophosphocholine and various concentrations of divalent cations. Error bars indicate the standard deviation (based on three independent experiments).

In order to assess the biochemical properties and physiological relevance of the putative glycerophosphodiesterases, their corresponding genes, *glpQ* and *mpn566*, were cloned into the expression vector pGP172, thus allowing a fusion of the proteins to an N-terminal *Strep*-tag facilitating purification. The recombinant proteins were purified and the activities were first determined using glycerophosphocholine (GPC) as the substrate and a set of divalent cations. As shown in Figure **2**, purified GlpQ was active against GPC, and the activity was highest in the presence of magnesium ions (10 mM). Manganese and zinc ions did also support activity, although to a lesser extent (Figure **2**). In contrast, the enzyme was inactive in the presence of calcium and cobalt ions (data not shown). The activity assay with purified MPN566 revealed no activity with GPC, irrespective of the cation present in the assay (data not shown). We did also test the activity of both proteins with glycerophosphoethanolamine and glycerophosphoglycerol. However, neither protein was active with any of these substrates. Thus, our data demonstrate that GlpQ is active as a glycerophosphodiesterase, whereas MPN566 does not exhibit such an activity.

The two proteins GlpQ and MPN566 share ∼58% identical residues. Thus, it seems surprising that MPN566 was inactive in the enzymatic assay. However, several residues that are known to be important for the activity of glycerophosphodiesterases are conserved in GlpQ but not in MPN566 (Figure **S1**). These residues include Trp-36 and Glu-38 as well as the conserved HD motif (Asn-51 and Leu-52 in MPN566) and Phe-110. Interestingly, a similar arrangement with two GlpQ-like proteins is also observed in *M. genitalium*, and as in *M. pneumoniae*, one protein has all the conserved residues characteristic for glycerophosphodiesterases, whereas the second protein has similar deviations from the consensus as MPN566 (Figure **S1**). In order to test whether a restoration of the conserved residues would also convert MPN566 to a biologically active glycerophosphodiesterase, we replaced the five amino acids that differ from the consensus by those residues present in GlpQ. The resulting mutant allele was cloned into pGP172, and purification attempted. Unfortunately, this protein was highly unstable and purification was impossible.

### Construction of *glpQ* and *mpn566* mutants

The analysis of mutants is one of the most powerful tools for studying gene functions and bacterial physiology. The isolation of desired *M. pneumoniae* mutants became possible only recently by the introduction of the “Haystack mutagenesis” [27]. To get more insights into the physiological role of the glycerophosphodiesterases GlpQ and its paralog MPN566, we attempted to isolate mutants affected in the corresponding genes.

**Figure 3:**
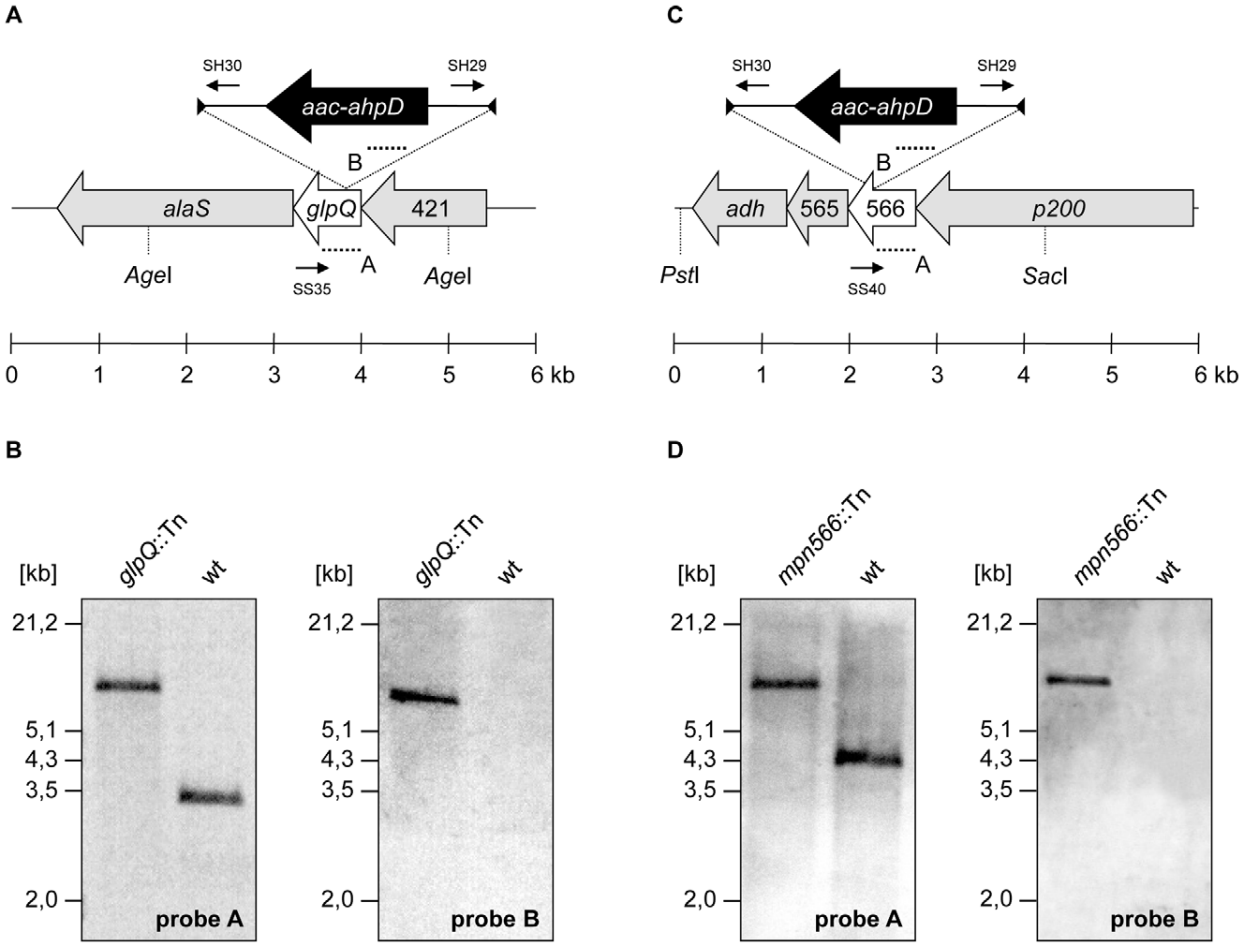
Isolation of *M. pneumoniae* glycerophosphodiesterases transposon insertion mutants. (A, C) Schematic representation of the genomic region surrounding the *glpQ* and *mpn566* gene (both designated as glycerophosphodiesterases) in *M. pneumoniae* and site of the transposon insertion in the knockout strains GPM81 and GPM82, respectively. The annealing sites of oligonucleotides used for the determination of the transposon insertion site are indicated by small arrows. Probes A and B hybridizing to internal fragments of the glycerophosphodiesterases and the *aac-ahpD* genes are depicted as dotted lines. (B, D) Southern blot analysis to confirm the single insertion of the mini-transposon into the *glpQ* and *mpn566* gene of the strains GPM81 and GPM82, respectively. Chromosomal DNAs of the wild type and both glycerophosphodiesterase mutants were digested as indicated. Blots were hybridized with the respective glycerophosphodiesterase-specific probe (left) and a probe hybridizing to the *aac-ahpD* gene of the mini-transposon (right).

The strategy of “Haystack mutagenesis” is based on an ordered collection of pooled random transposon insertion mutants that can be screened for junctions between the transposon and the gene of interest due to transposon insertion. The 64 pools were used in a PCR to detect junctions between the *glpQ* or *mpn566* genes and the mini-transposon using the oligonucleotides SS35 and SS40 (for the respective genes) and SH30 (for the mini-transposon) [28] (Figure **3**). Positive signals were obtained for both genes. From pools that gave a positive signal, colony PCR with the 50 individual mutants resulted in the identification of the desired *glpQ* and *mpn566* mutants. The presence of the transposon insertion in both genes was verified by Southern blot analysis (Figure **3**). To test whether these strains contained only unique transposon insertions, we did another Southern blot using a probe specific for the *aac-aphD* resistance gene present on the mini-transposon. As shown in Figure **3**, only one band hybridizing with this probe was detected in each strain, moreover, this fragment had the same size as the *Age*I or *Pst*I/*Sac*I fragment hybridizing to the *glpQ* and *mpn566* probe, respectively (Figure **3**). The isolated *glpQ* and *mpn566* mutant strains were designated GPM81 and GPM82. The position of the transposon insertion in the two genes was determined by DNA sequencing. The *glpQ* gene was disrupted between nucleotides 517 and 518, resulting in a truncated protein of 172 amino acids with one additional amino acid and the following stop codon encoded by the inserted mini-transposon. The disruption of the *mpn566* gene was located between nucleotides 157 and 158, resulting in a truncated protein of 52 amino acids with one additional amino acid and the following stop codon.

### Contributions of GlpQ and MPN566 to growth and motility

**Figure 4:**
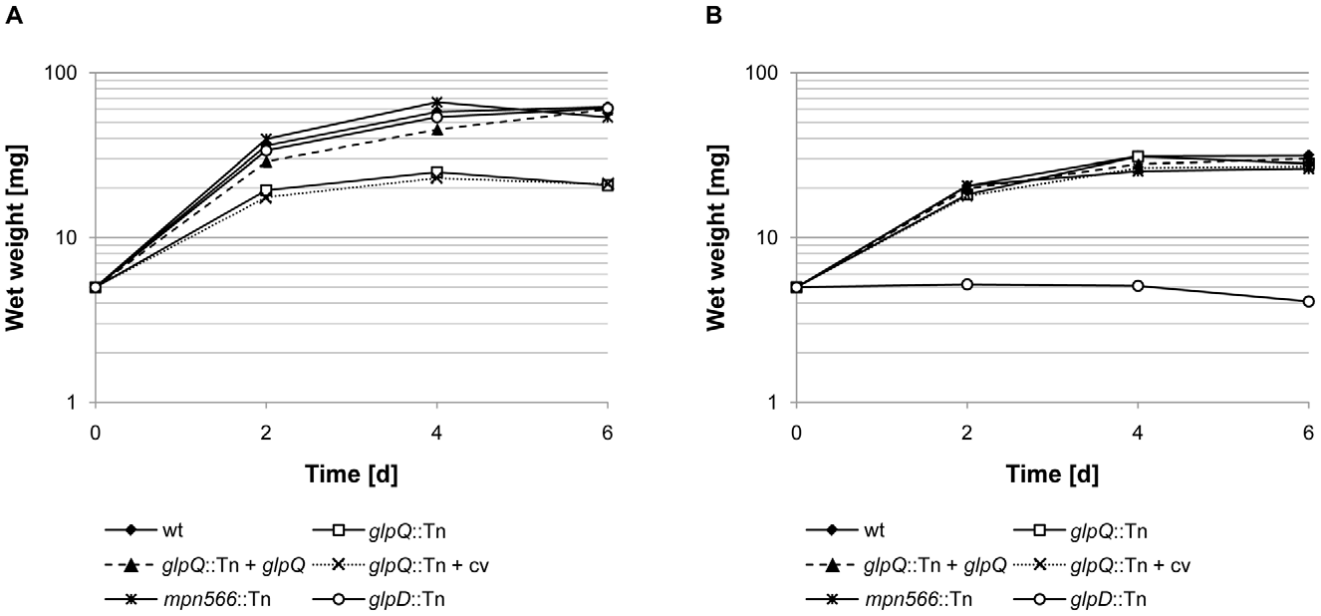
Growth of *M. pneumoniae* in modified Hayflick medium containing different carbon sources. One hundred milliliters of medium were inoculated with 5 mg of the relevant *M. pneumoniae* cells and incubated for up to six days at 37°C in 150-cm^2^ cell culture flasks. The following strains were used: wild type (wt), *glpQ*::Tn, *mpn566*::Tn, and *glpD*::Tn, *glpQ*::Tn + *glpQ* (complemented mutant) and *glpQ*::Tn + cv (control strain carrying the empty vector used for complementation). Glucose (A) and glycerol (B) were added to a final concentration of 1% (wt/vol). Attached cells were collected by scraping and growth was monitored by determination of the wet weight of the cell pellets. All measurements were done three times. Results are from a representative experiment.

First, we compared the ability of the wild type strain and the two mutant strains to utilize glucose and glycerol as the single carbon sources (Figure **4**). As an additional control, we used the *glpD* mutant strain GPM52. This strain is defective in glycerol-3-phosphate oxidase and therefore unable to utilize glycerol as the only carbon source [10]. As shown in Figure **4A**, the wild type and the *glpD* and *mpn566* mutant strains grew well with glucose. In contrast, the *glpQ* mutant GPM81 grew more slowly and did not reach the final biomass as compared to the other strains. As reported previously, the wild type strain exhibited very slow growth with glycerol as the only carbon source [29]. In this respect, the *glpQ* and *mpn566* mutants were indistinguishable from the wild type. As reported previously, the *glpD* mutant strain did not grow at all in glycerol-containing medium [10]. In conclusion, the active glycerophosphodiesterase GlpQ is required for maximal growth in the presence of glucose, whereas its absence does not interfere with the slow growth in the presence of glycerol.

Since the disruption of *glpQ* affected the growth properties of the bacteria, we wondered whether this might reflect changes in cell morphology and in the movement of the bacteria. The morphology of the wild type and mutant bacteria was analyzed by scanning electron microscopy, and no differences were detected (Figure **S2**). An analysis of the gliding velocities of the three strains revealed that the wild type strain glided with a velocity of 0.32±0.09 µm/s, whereas the *glpQ* and *mpn566* mutants exhibited velocities of 0.2±0.08 µm/s and 0.3±0.1 µm/s, respectively (Videos **S1**, **S2**, and **S3**). Thus, the active glycerophosphodiesterase GlpQ is required for full gliding velocity of the bacteria.

### Implication of GlpQ and MPN566 in hydrogen peroxide production and cytotoxicity

**Figure 5:**
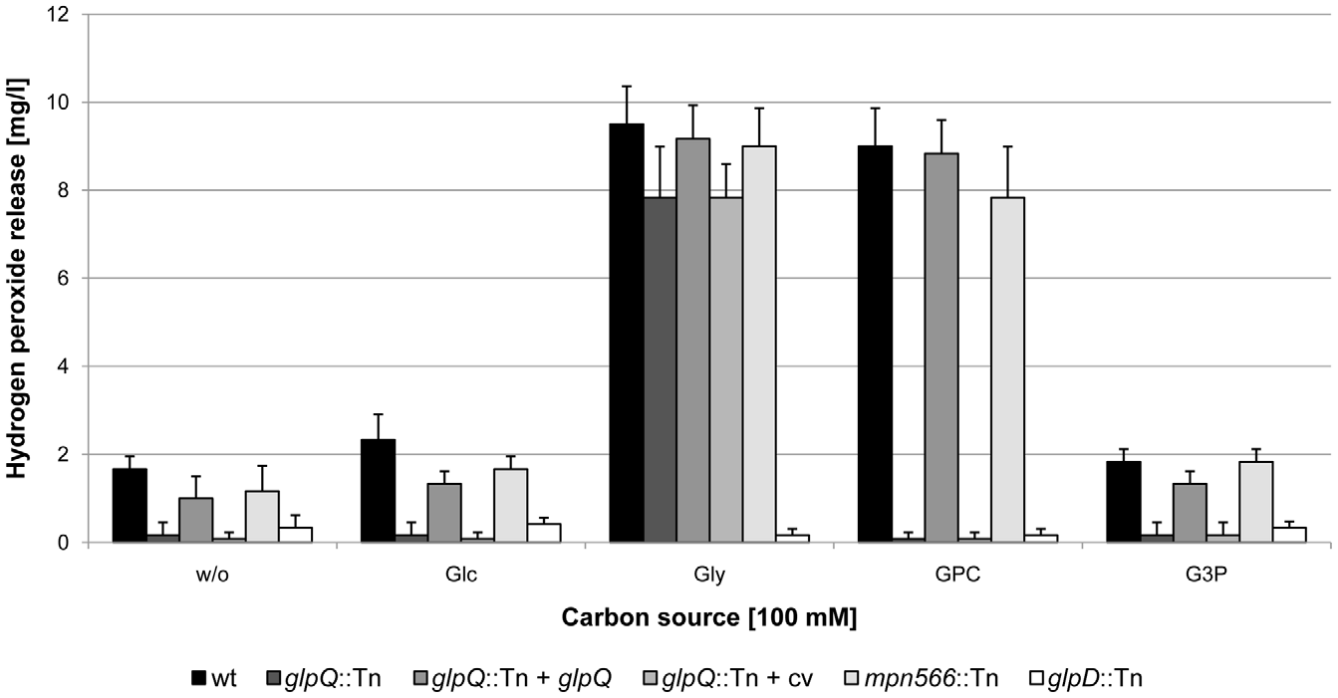
Examination of *M. pneumoniae* hydrogen peroxide release. Hydrogen peroxide production of *M. pneumoniae* mutant strains was measured in the presence of different carbon sources (100 µM) after 2 h. The following strains were used: wild type (wt), *glpQ*::Tn, *mpn566*::Tn, and *glpD*::Tn, *glpQ*::Tn + *glpQ* (complemented mutant) and *glpQ*::Tn + cv (control strain carrying the empty vector used for complementation). Error bars indicate standard deviation (based on three independent experiments). G3P, glycerol-3-phosphate; GPC, glycerophosphocholine; Glc, glucose; Gly, glycerol; w/o, without addition of any carbon source.

The utilization of glycerol or glycerophosphodiesters results in the generation of hydrogen peroxide, the major cytotoxic product of *M. pneumoniae*. We asked therefore whether the *glpQ* and *mpn566* disruptions would affect hydrogen peroxide formation and if so, whether it also affects cytotoxicity. Hydrogen peroxide formation was assayed in *M. pneumoniae* cultures that contained glucose, glycerol, GPC, glycerol-3-phosphate or no carbon source. In the absence of an added carbon source, neither the wild type strain nor the mutants formed substantial amounts of hydrogen peroxide (Figure **5**). It is interesting to note that the wild type and the *mpn566* mutant formed some hydrogen peroxide even in the absence of any added carbon source. This might result from the presence of low concentrations of phospholipids in the medium. Similarly, essentially no hydrogen peroxide was produced in the presence of glucose. If glycerol was available, maximal hydrogen peroxide formation (9.5 mg/l) was observed in the wild type strain. In the *glpD* mutant that served as a control, no hydrogen peroxide was formed. This is in good agreement with previous reports on the increase of hydrogen peroxide generation in the presence of glycerol and its dependence on a functional glycerol-3-phosphate oxidase [10]. The hydrogen peroxide production in the *glpQ* and *mpn566* mutants was similar to that observed in the wild type strain. This result reflects that the metabolite glycerol is downstream from the glycerophosphodiesterase activity. In the presence of GPC, the wild type strain produced similar amounts of hydrogen peroxide (9 mg/l) as in the presence of glycerol. In contrast, no hydrogen peroxide formation was detected for the *glpQ* mutant GPM81, whereas the disruption of *mpn566* did not have any effect on the production of hydrogen peroxide (Figure **5**). This result is in good agreement with the enzymatic activities of the two proteins: GlpQ is the only active glycerophosphodiesterase in *M. pneumoniae*, and no glycerol-3-phosphate, the substrate of GlpD, can be formed in its absence, whereas MPN566 is dispensable for the utilization of GPC. We also tested the ability of the *M. pneumoniae* strains to form hydrogen peroxide in the presence of glycerophosphoethanolamine and glycerophosphoglycerol. These compounds did not stimulate hydrogen peroxide in any of the strains tested (data not shown). This is in excellent agreement with the result of the enzyme assay that suggested that neither GlpQ nor MPN566 is able to degrade these substances. Finally, we tested whether hydrogen peroxide was formed in the presence of glycerol-3-phosphate. As shown in Figure **5**, no significant formation of hydrogen peroxide was observed in any of the strains tested. This suggests that the uptake of glycerol-3-phosphate is rather inefficient.

**Figure 6:**
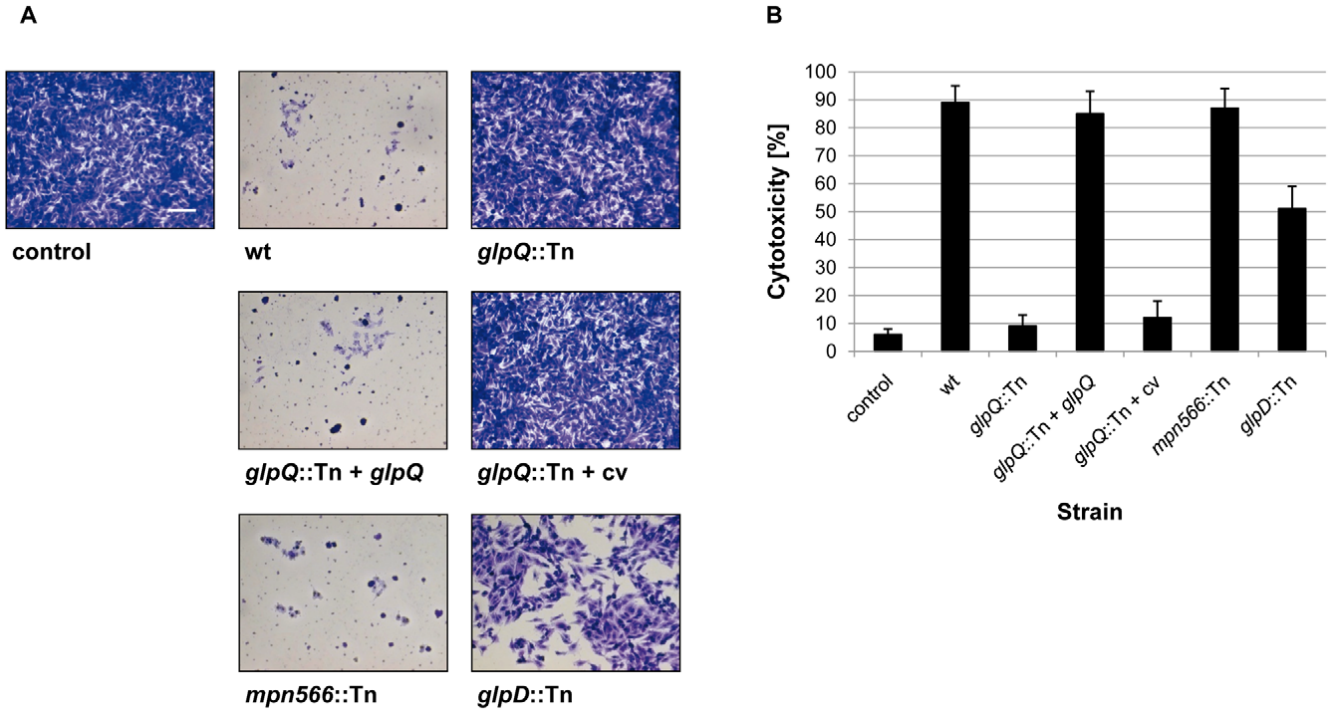
Cytotoxicity of *M. pneumoniae* toward HeLa cell cultures. (A) Infection assay to verify cytotoxic effects of *M. pneumoniae glpQ*::Tn and *mpn566*::Tn mutant strains. HeLa cells were infected with *M. pneumoniae* wild type (wt), *glpQ*::Tn, and *mpn566*::Tn mutant cells. As control served two HeLa cell cultures: One without addition of any *M. pneumoniae* cells and another after infection with *glpD*::Tn mutant strain [10]. Moreover, cytotoxicity of the complemented mutant (*glpQ*::Tn + *glpQ*) and of the mutant carrying the empty vector (*glpQ*::Tn + cv) was tested. After four days, HeLa cell cultures were stained with crystal violet and photographed. All pictures are shown at the same magnification. Scale bar, 0.1 mm. (B) Quantification of cytotoxicity caused by *M. pneumoniae* in HeLa cell cultures. HeLa cells were infected with different *M. pneumoniae* strains or left uninfected (negative control). LDH release of HeLa cell cultures after 2 h of infection was used as an index of cytotoxicity. Cytotoxicity was calculated as the percentage of total LDH release after complete cell lysis. Error bars indicate standard deviation (based on three independent experiments).

To assess the cytotoxicity of the different *M. pneumoniae* strains, we infected confluently grown HeLa cell cultures with *M. pneumoniae* cells (multiplicity of infection: 2). The cytotoxicity of the mutants was compared to that of the wild type strain and *M. pneumoniae* GPM52 that is affected in *glpD*. As shown in Figure **6**, the HeLa cells had undergone nearly complete lysis after four days upon infection with wild type *M. pneumoniae* (cytotoxicity of 89%). As observed previously, the *glpD* mutant GPM52 has a reduced cytotoxicity (51%) resulting in a large portion of viable cells after infection [10]. For the *glpQ* mutant GPM81, nearly all HeLa cells had survived the infection suggesting that GlpQ is essential for cytotoxicity. In contrast, cytotoxicity induced by the *mpn566* mutant strain GPM82 was equivalent to that of the wild type strain (Figure **6**). These data clearly demonstrate that the active glycerophosphodiesterase GlpQ is required for host cell damage, whereas MPN566 is not. Moreover, they support the assumption that hydrogen peroxide formation is the major factor that contributes to host cell damage.

### Complementation of the *glpQ* mutation

In order to exclude the possibility that the phenotypes observed with the *glpQ* mutant are due to a polar effect on the downstream *alaS* gene, we compared the *alaS* transcript levels in the wild type strain and the *glpQ* mutant. We observed fourfold increased amounts of *alaS* mRNA levels in the *glpQ* mutant, most likely due to the presence of a strong promoter in the transposon (data not shown). The expression of the *alaS* gene in the *glpQ* mutant strongly suggests that the observed phenotypes are the result of the *glpQ* disruption rather than of a polar effect. However, to provide unequivocal evidence for the implication of GlpQ in the growth phenotype as well as in hydrogen peroxide production and cytotoxicity, we performed a complementation assay. For this purpose, the *M. pneumoniae glpQ* gene with its own promoter was cloned into the integrative vector pMTnTetM438 and introduced into the chromosome of the *glpQ* mutant GPM81 (for details, see “Materials and Methods”). The resulting complementation strain GPM92 and the isogenic *glpQ* mutant GPM93 carrying the empty vector integrated into the chromosome were analysed for growth in the presence of glucose and glycerol, for hydrogen peroxide formation and for cytotoxicity. As shown in Figure **4A**, the complemented mutant grew in the presence of glucose as the wild type strain. In contrast, the control strain carrying the empty vector grew slowly in the presence of glucose and was in this respect indistinguishable from the original *glpQ* mutant. These data clearly establish that the growth defect of the *glpQ* mutant in Hayflick medium containing glucose is a specific result of the *glpQ* inactivation. Similar results were observed for hydrogen peroxide production. Ectopic expression of the *glpQ* gene in the mutant strain restored the wild type phenotype, *i.e.* strong hydrogen peroxide formation in the presence of GPC. Again, the empty vector did not alter the phenotype of the *glpQ* mutant (see Figure **5**). Finally, we assessed the cytotoxicity of the complemented strain towards HeLa cells. As one would expect from the restoration of hydrogen peroxide production upon complementation, the complemented mutant GPM92 was toxic for the HeLa cells, whereas the mutant carrying the control vector was not (Figure **6**). In conclusion, ectopic expression of *glpQ* complemented all mutant phenotypes thus demonstrating, that the active glycerophosphodiesterase GlpQ is indeed essential for hydrogen peroxide production in the presence of the major substrate glycerophosphocholine and for cytotoxicity of *M. pneumoniae*.

### The role of GlpQ in gene expression

As reported above, the *glpQ* mutant exhibits multiple phenotypes related to motility, metabolism, and pathogenicity. We asked therefore whether some of the effects are due to changes in the proteome of the *glpQ* mutant GPM81. To answer this question, we compared the total protein profiles of the wild type strain and the *glpQ* and *mpn566* mutants, GPM81 and GPM82, respectively, after growth in glucose and glycerol. While the protein patterns in the *mpn566* mutant were indistinguishable from the wild type strain under both conditions, several differences were noted for the *glpQ* mutant.

To identify those proteins that exhibit altered accumulation in the *glpQ* mutant, the total proteins of the wild type and the *glpQ* mutant strains were identified by mass spectrometry. For the protein extracts from glucose-grown cells, 532 different proteins were identified. This corresponds to about 77% of the theoretical proteome of *M. pneumoniae*. In the presence of glycerol, 473 proteins corresponding to 69% of the theoretical proteome were identified. The differences in protein expression between glucose- and glycerol-grown cells as well as proteins that could not be detected at all are summarized in Tables **S1** and **S2**. A detailed list of the differences of the protein profiles between the wild type strain and the *glpQ* mutant is presented in Tables **S3** and **S4**. As expected, the GlpQ protein was detected in the protein extracts of the wild type strain but not in those of the *glpQ* mutant strain. In glucose-grown cells, 33 and 21 proteins were in elevated and reduced amounts, respectively, in the *glpQ* mutant. The strongest increase was observed for the glycerol facilitator GlpF and the uncharacterized lipoprotein MPN162. A strongly reduced accumulation was observed for the lipoprotein MPN506. In the presence of glycerol, five induced and five repressed proteins were detected (see Table **S4**). Five proteins were subject to a identical regulation under both conditions (Table **1**).

**Table 1:** Proteins with GlpQ-dependent expression pattern.

			Fold-change in the presence of glucose^a^		Fold-change in the presence of glycerol^a^	
Locus name	Protein name	Protein function	Protein level	Transcript level	Protein level	Transcript level
MPN043	GlpF	Glycerol uptake facilitator	10.79±1.06	5.69±0.36	5.83±0.76	3.32±0.34
MPN162	-	Uncharacterized lipoprotein	9.96±0.97	5.23±0.23	5.41±0.83	2.81±0.47
MPN284	-	Uncharacterized lipoprotein	ns	0.25±0.05	0.36±0.02	0.35±0.04
MPN433	CbiO	Metal ion ABC transporter	ns	7.56±0.39	5.66±0.49	2.92±0.24
MPN506	-	Uncharacterized lipoprotein	0.10±0.03	0.07±0.05	0.18±0.04	0.16±0.02

a Fold-change cut off ≥2.0 and ≤0.5, respectively (*glpQ* mutant strain vs. wild type). Abbreviation: ns, no significant difference.

For detailed information on proteome and transcript changes in the *glpQ* mutant GPM81, see Tables S3 and S4.

**Figure 7:**
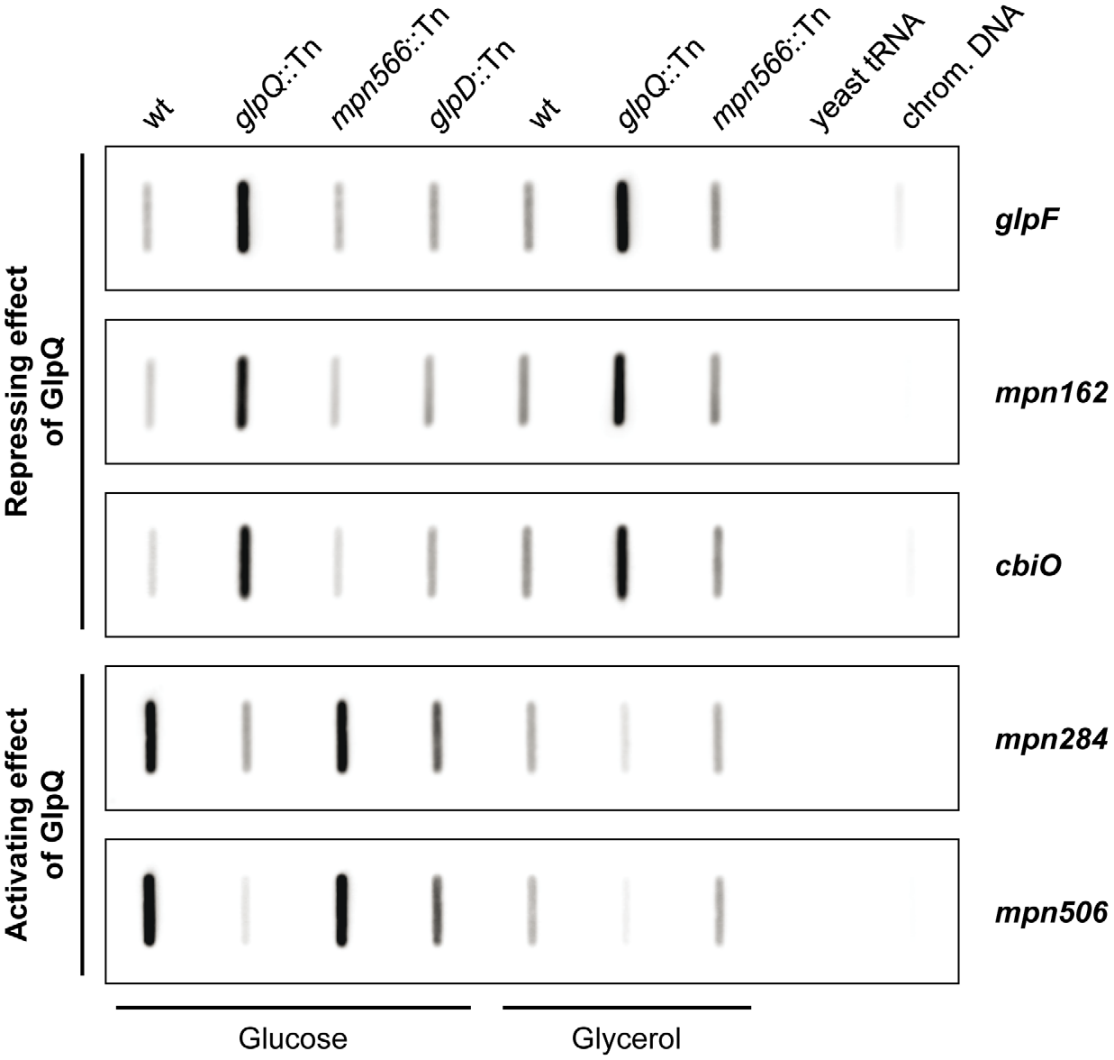
Transcription analysis of GlpQ-dependent genes in *M. pneumoniae*. Slot blots were performed with whole RNA extracts of *M. pneumoniae* wild type (wt), *glpQ*::Tn, *mpn566*::Tn, and *glpD*::Tn (control) mutant strains grown in modified Hayflick medium containing either glucose or glycerol as sole carbon source (1% [wt/vol]). A dilution series of RNA extracts was blotted onto a positively charged nylon membrane and probed with a DIG-labeled riboprobe specific for an internal part of a particular open reading frame. Names of riboprobes are given next to each blot. Signals obtained with 1 µg of RNA are shown. Yeast tRNA and *M. pneumoniae* chromosomal DNA served as controls. For detailed information on differences of transcript levels see Table **1**, **S3**, and **S4**.

It has been shown before that changes at the proteome level may result from altered gene expression or from changes in protein stability [30], [31]. Therefore, we studied the expression of the genes corresponding to the most prominently regulated proteins and of genes encoding potential regulators, transport systems and potential pathogenicity factors. For this purpose, we isolated RNA from cultures grown in modified Hayflick medium supplemented with glucose and performed slot blot analyses (Figures **7** and **S3**).

These studies demonstrated that the regulation of the glycerol facilitator GlpF and the lipoproteins MPN162 and MPN506 occurs at the level of transcription (Table **1**). Moreover, our results confirmed the higher expression of *glpF* and *mpn162* and the repression of *mpn506* in the *glpQ* mutant. For the other proteins that were induced in the presence of glucose, with exception of *plsC* and *mpn566* (nearly two-fold higher transcript levels), similar accumulation of the mRNAs compared to the protein amount was observed (Table **S3** and Figure **S3**). In contrast, for the proteins that were present in reduced amounts in glucose-grown cells, no changes of the corresponding mRNAs were observed for all transport proteins. Interestingly, the lipoprotein MPN083 showed a similar pattern at the level of transcription as the induced proteins and the ribonucleoside-diphosphate reductase (encoded by *nrdFIE*) was the only protein with reduced mRNA amounts, however changes in transcript level were not significant (Table **S3** and Figure **S3**).

### Identification of a potential target site for GlpQ-dependent regulation

**Figure 8:**
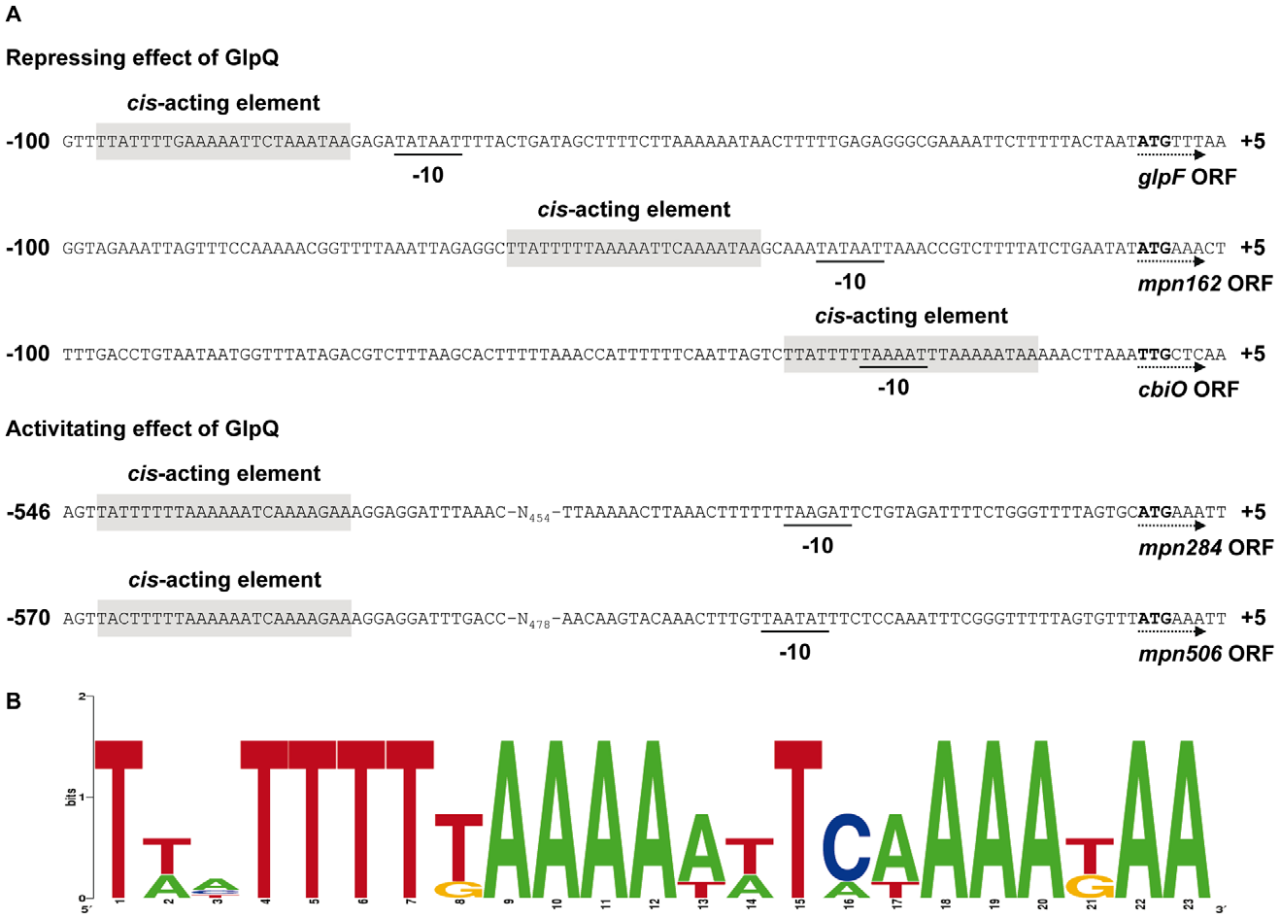
Transcriptional organization of GlpQ-dependent genes in *M. pneumoniae*. (A) Nucleotide sequence of promoter regions of *M. pneumoniae glpF* (*mpn043*), *mpn162*, *cbiO* (*mpn433*), *mpn284*, and *mpn506* genes. Promoter sequences are numbered relative to the 5’ end. Directions of the open reading frames (ORF) are indicated by dotted arrows and predicted ATG/TTG start codons are highlighted by bold type. The respective -10 motifs are underlined. GlpQ-dependent *cis*-acting elements (palindromic DNA motifs) are indicated by grey shading. (B) Consensus sequence of the GlpQ-dependent palindromic DNA motif in *M. pneumoniae*. The sequence logo was created using WebLogo v2.8.2 [50] based on all five palindromic DNA motifs mentioned in A.

The proteome and transcription analyses identified three genes that are significantly regulated - either induced or repressed - in a GlpQ-dependent manner. An inspection of the upstream region of these genes revealed the presence of a common palindromic DNA motif (Figure **8**). To exclude the possibility that this motif is randomly distributed in the genome of *M. pneumoniae* because of the extremely AT-rich consensus sequence, we tested its presence in the genome using the GLAM2SCAN algorithm [32]. In nine cases (matching score cut-off ≥30), this potential motif was located upstream of open reading frames, among them the three genes mentioned above. Therefore, the expression of the remaining six genes was tested by slot blot analysis, and for two of these genes, *cbiO* and *mpn284*, a significant accumulation and reduction of the mRNA, respectively, was observed (data not shown; Figure **7**). Interestingly, the corresponding proteins, a subunit of a putative metal ion ABC transporter CbiO and the uncharacterized lipoprotein MPN284 were found to be present in higher or lower amounts in the *glpQ* mutant in glycerol-grown cell. Thus, there is a very good agreement between the regulatory effect of GlpQ at the proteome level, the regulation at the level of transcription, and the presence of the *cis*-acting element.

## Discussion

Two cytotoxicity factors are known in *M. pneumoniae*: The formation of hydrogen peroxide and the CARDS toxin that possesses ADP-ribosyltransferase activity [11], [33]. This work establishes that the glycerophosphodiesterase GlpQ of *M. pneumoniae* is essential for cytotoxicity of these bacteria. This is in excellent agreement with previous reports that carbon metabolism is intimately linked to virulence in pathogenic bacteria, including *M. pneumoniae* and other mollicutes [1], [2], [8]. The utilization of glycerol and phospholipids plays a particularly important role in the virulence of *Mycoplasma* species: Hydrogen peroxide, the major cytotoxic substance produced by these bacteria, is generated as a product of glycerol metabolism, and both *glpD* and *glpQ* mutants are severely affected in pathogenicity [10, this work]. In *M. mycoides*, pathogenicity is associated with the presence of a highly efficient ABC transporter for glycerol. Non-pathogenic strains of *M. mycoides* rely on the less efficient glycerol facilitator for glycerol uptake [34].

In *M. pneumoniae*, GlpQ is not only important for virulence but also for growth in the commonly used medium in the laboratory, *i.e.* Hayflick medium with glucose as the added carbon source (see Figure **4A**). This observation is in good agreement with a recent analysis of the *M. pneumoniae* metabolism that suggested that glycerol is essential for growth of *M. pneumoniae* [35]. Accordingly, no difference between the wild type strain and the *glpQ* mutant was observed during growth in the presence of glycerol (see Figure **4B**). Therefore, it is tempting to speculate that some glycerophosphodiesters in the Hayflick medium support growth.

In addition to GlpQ, *M. pneumoniae* encodes a second paralogous protein. However, as shown in this work, this protein does not exhibit enzymatic activity nor does the inactivation of the corresponding gene (*mpn566*) cause any detectable phenotype. This lack of detectable activity of MPN566 is easily explained by the lack of conservation of amino acid residues that are essential for the activity as a glycerophosphodiesterase. Interestingly, a very similar arrangement with two *glpQ*-like genes is also present in *M. genitalium* and *Mycoplasma alligatoris*. Based on the conservation of the catalytically important residues (see Figure **S1**), there is an active and an inactive enzyme in *M. genitalium*, as observed here for *M. pneumoniae*. In *M. alligatoris*, both potential glycerophosphodiesterases contain all the important amino acids suggesting that both proteins are enzymatically active. It is tempting to speculate that the possession of two active glycerophosphodiesterases is related to the fact that *M. alligatoris* is the only mollicute that obligatorily causes fatal infections [36]. In the syphilis spirochaete, *Treponema pallidum*, one *glpQ*-like gene is present; however, the encoded protein is not active as a glycerophosphodiesterase. Again, the inactivity is most likely caused by the lack of conservation of functionally important amino acids [37], [38]. The presence of inactive GlpQ-like proteins in several pathogens, including a spirochaete and *M. genitalium*, the bacterium with the smallest genome, suggests that these proteins have other functions that have yet to be identified. Unfortunately, the experiments reported in this study did not give any hints as to a putative function of MPN566.

Many proteins have activities in addition to their primary functions. On one hand, this allows gene duplication and specialization to non-related functions of similar proteins. On the other hand, a protein may acquire a second useful activity and act as a so-called moonlighting protein [39]. The former is very common and might apply to the putative functional specialization of GlpQ and MPN566. In contrast, the latter phenomenon is true for all trigger enzymes that measure the availability of their respective metabolites and transduce this information to the regulatory machinery of the cell. In mammals, a glycerophosphodiesterase controls the development of skeletal muscles independent from its enzymatic activity [40]. Our results suggest that GlpQ might also have such a second activity. Indeed, the expression of the glycerol facilitator GlpF, a lipoprotein, and the ATP-binding subunit of a metal ion ABC transporter are strongly overexpressed in the *glpQ* mutant, whereas two uncharacterized lipoproteins are less expressed in the mutant. Interestingly, the genes that appear to be repressed by GlpQ are more strongly transcribed in the presence of glycerol as the carbon source (as compared to glucose). In contrast, the two lipoprotein genes *mpn284* and *mpn506* that require GlpQ for expression are only weakly expressed in the presence of glycerol, but they are strongly induced if glucose is used as the carbon source. These observations might be explained as follows: In the presence of glucose, only little glycerol or glycerol-3-phosphate (the product of the reaction catalyzed by GlpQ) is present in the cell. Free GlpQ might then directly bind DNA or trigger the DNA-binding activity of another, yet unknown transcription factor, resulting in repression or activation of the two sets of genes. In the presence of glycerol, glycerol-3-phosphate would be formed due to the activity of glycerol kinase, and this metabolite might then prevent GlpQ from its regulatory activity. As a result, those genes that are subject to GlpQ-dependent repression (*glpF*, *mpn162*, and *cbiO*) are stronger expressed than in the presence of glucose, whereas the GlpQ-activated genes (*mpn284*, *mpn506*) would be less expressed. Finally, in the *glpQ* mutant, the former set of GlpQ-repressed genes is highly constitutively expressed, and only a very low level of transcription can be detected for the two GlpQ-dependent lipoprotein genes. Since glycerol-3-phosphate is the product of the glycerophosphodiesterase reaction, this metabolite is an excellent candidate for detection by GlpQ. Moreover, the *glpQ* gene is constitutively expressed and the GlpQ protein was detected in *M. pneumoniae* cells irrespective of the carbon source used in similar amounts in this study [41, this study]. Thus, GlpQ is available in the cell under all conditions to cause regulation. In a recent study on the phosphoproteome of *M. pneumoniae*, phosphorylation of GlpQ was observed [42]; however, no precise phosphorylation site could be detected and predicted, respectively. Therefore, the functional relevance of this modification remains unknown so far.

As observed for several other transcription regulators and trigger enzymes, GlpQ exerts both an activating and repressing effect on gene expression. The location of the putative *cis*-acting element correlates perfectly with the regulatory effect: Those genes that seem to be repressed by GlpQ-dependent manner have this element overlapping or in the very close vicinity of the -10 region of the promoters. This element is the only conserved promoter element in *M. pneumoniae* and it is sufficient for transcription initiation [30], [41], [43]. Binding of GlpQ or of a transcription factor that is controlled by GlpQ would prevent a productive interaction with RNA polymerase and therefore cause transcription repression. On the other hand, the *cis*-acting elements that may be involved in the regulation of the GlpQ-activated genes are located upstream of the promoters. This is usually the case for binding sites of transcription activators and fits perfect with the observed regulation.

Our future work will focus on the elucidation of the mechanism(s) by which GlpQ controls gene expression. Moreover, we will address the functions of the lipoproteins that are subject to glycerol- and GlpQ-dependent regulation.

## Materials and Methods

### Bacterial strains and growth conditions

The *M. pneumoniae* strains used in this study were *M. pneumoniae* M129 (ATCC 29342) in the 32nd broth passage, and its isogenic mutant derivatives GPM52 (*glpD*::mini-Tn, Gm^R^) [10], GPM81 (*glpQ*::mini-Tn, Gm^R^), and GPM82 (*mpn566*::mini-Tn, Gm^R^). *M. pneumoniae* was grown at 37°C in 150 cm^2^ tissue culture flasks containing 100 ml of modified Hayflick medium as described previously [29]. Carbon sources were added to a final concentration of 1% (w/v). Growth curves were obtained by determining the wet weight of *M. pneumoniae* cultures as described previously [29]. Strains harboring transposon insertions were cultivated in the presence of 80 µg/ml gentamicin and/or 2 µg/ml tetracycline as required. *Escherichia coli* DH5α and BL21(DE3)/pLysS [44] were used as host for cloning and recombinant protein expression, respectively.

The sequences of the oligonucleotides used in this study are listed in Table S5.

### Construction of a *glpQ* complementation strain

To achieve complementation of the *glpQ* mutant, we constructed strain GPM92 as follows: The *M. pneumoniae glpQ* gene including its own promoter was amplified using the primer pair SS245/SS267. The PCR product was digested with *Eco*RI and *Xho*I and cloned between the *Eco*RI/*Sal*I sites of the integrative plasmid pMTnTetM438 [45]. The resulting plasmid, pGP695, was introduced by electroporation into the genome of the *M. pneumoniae glpQ* mutant GPM81. As a control, we transformed GPM81 with the empty vector pMTnTetM438. The resulting strain was *M. pneumoniae* GPM93. To exclude multiple insertions of the integrative plasmids in the two constructed strains, we performed Southern blot analyses with both mutants using a probe specific for the tetracycline resistance gene. In both cases, unique insertion events were detected.

*M. pneumoniae* chromosomal DNA was prepared as described previously [28]. Finally, digests of chromosomal DNA were separated using 1% agarose gels and transferred onto a positively charged nylon membrane (Roche Diagnostics) [44] and probed with Digoxigenin labeled riboprobes obtained by *in vitro* transcription with T7 RNA polymerase (Roche Diagnostics) using PCR-generated fragments as templates. Primer pairs for the amplification of *glpQ, mpn566*, *aac-ahpD,* and *tet* gene fragments were SS42/SS43, SS44/SS45, SH62/SH63, and SS272/SS273, respectively (Table **S5**). The reverse primers contained a T7 RNA polymerase recognition sequence. *In vitro* RNA labeling, hybridisation and signal detection were carried out according to the manufacturer’s instructions (DIG RNA labeling Kit and detection chemicals; Roche Diagnostics).

### Production and analysis of recombinant putative glycerophosphodiesterases

The *M. pneumoniae* genes encoding proteins similar to glycerophosphodiesterases (*glpQ* and *mpn566*) were amplified with chromosomal DNA as the template and the primer pairs SS34/SS35 and SS39/SS40, respectively. The PCR products were digested with *Sac*I and *Bam*HI and cloned into the expression vector pGP172 that allows the fusion of the target proteins to a *Strep*-tag at their N-terminus [46]. The resulting plasmids were pGP1018 and pGP1020. Since the *glpQ* gene contains three TGA codons that are recognized as stop codons in *E. coli*, these codons were replaced by TGG specifying tryptophan as in *M. pneumoniae*. For this purpose we applied the multiple mutation reaction [47] using the phosphorylated mutagenesis primers SS36, SS37, and SS38 and the external primers SS34 and SS35. The PCR product was digested and cloned into pGP172 as described above. The resulting expression vector was pGP1019. The plasmids pGP1019 and pGP1020 allowed the purification of the putative *M. pneumoniae* glycerophosphodiesterases (GlpQ and MPN566) carrying an N-terminal *Strep*-tag.

A mutant variant of MPN566 was obtained by the multiple mutation reaction using pGP1020 as the template and the phosphorylated mutagenesis primers SS192, SS193, and SS194 and the external primers SS39 and SS40. The PCR product was cloned into pGP172 as described above and the resulting plasmid was pGP661.

The putative glycerophosphodiesterases were overexpressed in *E. coli* BL21(DE3)/pLysS. Expression was induced by the addition of IPTG (final concentration 1 mM) to exponentially growing cultures (OD_600_ of 0.8). Cells were lysed using a french press (20.000 p.s.i., 138,000 kPa, two passes, Spectronic Instruments, UK). After lysis the crude extracts were centrifuged at 15,000 *g* for 60 min. The crude extract was passed over a Streptactin column (IBA, Göttingen, Germany). The recombinant proteins were eluted with desthiobiotin (IBA, final concentration 2.5 mM).

After elution the fractions were tested for the desired protein using 12% SDS-PAGE. Only fractions that contained the desired protein in apparent homogeneity (content of the specific protein >95%) were used for further experiments. The relevant fractions were combined and dialyzed overnight. Protein concentration was determined according to the method of Bradford using the Bio-Rad dye-binding assay where Bovine serum albumin served as the standard.

Glycerophosphodiesterase activity was measured in a coupled spectrophotometric assay as described previously [48]. The enzyme assay is based on the formation of glycerol-3-phosphate and the subsequent oxidation by the glycerol-3-phosphate dehydrogenase and the formation of NADH. Briefly, 5 µg of glycerophosphodiesterase were incubated with 20 U of rabbit muscle glycerol-3-phosphate dehydrogenase (Sigma) in a 0.9 M glycine-hydrazine buffer containing 0.5 mM glycerophosphodiester and 0.5 mM NAD^+^ in a volume of 1 ml. Divalent cations were added as indicated. NADH formation was determined photospectrometrically at 340 nm.

### Determination of *in vivo* hydrogen peroxide production

The hydrogen peroxide production in *M. pneumoniae* was determined using the Merckoquant peroxide test (Merck, Darmstadt, Germany) as previously described [10]. Briefly, growing cells were resuspended in assay buffer and after incubation for 1 h at 37°C, glucose, glycerol, glycerol-3-phosphate or glycerophosphodiesters (final concentration 100 µM) were added to one aliquot. An aliquot without any added carbon source served as the control. The test strips were dipped into the suspensions for 1 s and subsequently read.

### Preparation and analysis of whole cell extracts

Whole cell extracts of the different *M. pneumoniae* strains were prepared as described previously [31]. In order to analyze the complete proteome, 15 µg of the cell extracts were separated by one-dimensional 12% SDS-PAGE and the gels subsequently stained with Coomassie Brillant Blue R250 dye (Serva). For protein identification, each running lane was cut out into 15 pieces followed by a separate analysis by mass spectrometry. The proteome analyses were performed in triplicate.

Gel pieces were washed twice with 200 µl 20 mM NH_4_HCO_3_/30% (v/v) acetonitrile for 30 min, at 37°C and dried in a vacuum centrifuge (Concentrator 5301, Eppendorf). Trypsin solution (10 ng/µl trypsin in 20 mM ammonium bicarbonate) was added until gel pieces stopped swelling and digestion was allowed to proceed for 16 to 18 hours at 37°C. Peptides were extracted from gel pieces by incubation in an ultrasonic bath for 15 min in 20 µl HPLC grade water and transferred into micro vials for mass spectrometric analysis.

The tryptic digested proteins obtained from the one-dimensional SDS PAGE gel pieces were subjected to a reversed phase column chromatography (Waters BEH 1.7 µm, 100-µm i.d.×100 mm, Waters Corporation, Milford, Mass., USA) operated on a nanoACQUITY UPLC (Waters Corporation, Milford, Mass., USA). Peptides were first concentrated and desalted on a trapping column (Waters nanoACQUITY UPLC column, Symmetry C_18_, 5 µm, 180 µm × 20 mm, Waters Corporation, Milford, Mass., USA) for 3 min at a flow rate of 1 ml/min with 0.1% acetic acid. Subsequently the peptides were eluted and separated with a non-linear 80-min gradient from 5–60% acetonitrile in 0.1% acetic acid at a constant flow rate of 400 nl/min. MS and MS/MS data were acquired with the LTQ Orbitrap mass spectrometer (Thermo Fisher, Bremen, Germany) equipped with a nanoelectrospray ion source. After a survey scan in the Orbitrap (r = 30,000), MS/MS data were recorded for the five most intensive precursor ions in the linear ion trap. Singly charged ions were not taken into account for MS/MS analysis.

Tandem mass spectra were extracted using Sorcerer v3.5 (Sage-N Research). All MS/MS samples were analyzed using SEQUEST (Thermo Fisher Scientific, San Jose, CA, USA; version 2.7, revision 11). Database searching was performed against a target decoy database of *M. pneumoniae* with added common laboratory contaminant proteins. Cleavage specificity for full tryptic cleavage and a maximum of 2 missed cleavages was assumed. SEQUEST was run with a fragment ion mass tolerance of 1.00 Da and a parent ion tolerance of 10 ppm. Oxidation of methionine (+15.99492 Da) and phosphorylation of serine/threonine/tyrosine (+79.966331 Da) were specified in SEQUEST as variable modifications. Proteins were identified by at least two peptides applying a stringent SEQUEST filter (Xcorr vs. charge state: 1.8 for singly, 2.2 for doubly, 3.3 for triply, and 3.5 for higher charged ions). To address protein amount differences between the *M. pneumoniae* wild type and mutant strains, fold-changes were calculated by comparing number of assigned spectra for each protein (mutant *vs*. wild type strain).

### Analysis of mRNA amounts

Preparation of total *M. pneumoniae* RNA was done as previously described [29]. For slot blot analysis, serial twofold dilutions of the RNA extract in 10x SSC (2 µg–0.25 µg) were blotted onto a positively charged nylon membrane using a PR 648 Slot Blot Manifold (Amersham Biosciences). Equal amounts of yeast tRNA (Roche) and *M. pneumoniae* chromosomal DNA served as controls. DIG-labelled riboprobes were obtained by *in vitro* transcription from PCR products that cover ORF internal sequences using T7 RNA polymerase (Roche). The reverse primers used to generate the PCR products contained a T7 promoter sequence (Table **S5**). The quantification was performed using the Image J software v1.44c [49].

### HeLa cell cytotoxicity assay

Infection of HeLa cell cultures with *M. pneumoniae* cells was done as described previously [10], [31]. After four days upon infection, HeLa cells cultures were stained with crystal violet and photographed. Additionally, lactate dehydrogenase (LDH) release of HeLa cell cultures after 2 h of infection was used as an index of cytotoxicity. LDH release was measured with the CytoTox 96 Non-Radioactive Cytotoxicity Assay (Promega) according to the manufacturer’s instructions. Results are expressed as cytotoxicity calculated as the percentage of total LDH release after cell lysis with the lysis buffer provided in the kit. The cytotoxicity assays were performed in triplicate.

### Scanning electron microscopy

After growing of *M. pneumoniae* cultures in 5 ml volumes to mid-log, the cells were scraped off and passed ten times through a syringe. Then, 20 µl of this cell suspension were inoculated to 2 ml of Hayflick medium in a Lab-Tek chamber slide (Nunc). After growing cells to mid-log phase, the medium was removed and the cells were washed three times with PBS and fixed with 1% glutaraldehyde for 1 h. The samples were washed three times with PBS and then dehydrated sequentially with 30, 50, 70, 90, and 100% ethanol for 10 min each. Immediately, the critical point dried of samples was performed (K850 critical point drier; Emitech Ashfort, United Kingdom) and sputter coated with 20 nm of gold. Samples were observed using a Hitachi S-570 (Tokyo, Japan) scanning electron microscope.

### Microcinematography

After passing through a syringe cells grown in a 5 ml culture, 20 µl of disaggregated cells were inoculated to 2 ml of modified Hayflick medium including 3% gelatine in 14 mm glass bottom culture dishes plates (MatTek). Cell movement was examined at 37°C using a Nikon Eclipse TE 2000-E microscope, and images were captured at intervals of 2 s for a total of 2 min with a digital sight DS-SMC Nikon camera controlled by NIS-Elements BR software. Tracks from 50 individual motile cells corresponding to 2 min of observation and 2 separated experiments were analyzed to determine the gliding velocity and gliding motile patterns.

## Supporting Information

Figure S1 **Multiple sequence alignment of GlpQ and MPN566 from** ***M. pneumoniae*** **with orthologous glycerophosphodiesterases of other bacteria.** The multiple sequence alignment was performed using ClustalW (http://www.ch.embnet.org/software/ClustalW.html) and represented with BOXSHADE v3.21 (http://www.ch.embnet.org/software/BOX_form.html). Black shading indicates ≥80% identity and grey shading stands for ≥80% similarity. Amino acids that constitute to the strictly conserved active site structure are depicted by black arrows. The UniProtKB entry names of the aligned sequences are Y420_MYCPN (GlpQ, *M. pneumoniae*), Y566_MYCPN (MPN566, *M. pneumoniae*), Y293_MYCGE (MG_293, *M. genitalium*), Y385_MYCGE (MG_385, *M. genitalium*), D4XVJ7_9MOLU (MALL_0582, *M. alligatoris*), D4XVF1_9MOLU (MALL_0631, *M. alligatoris*), Q8XNB7_CLOPE (GlpQ, *Clostridium perfringens*), Q8XJ84_CLOPE (GlpQ, *C. perfringens*), YHDW_BACSU (YhdW, *B. subtilis*), GLPQ_BACSU (GlpQ, *B. subtilis*), YQIK_BACSU (YqiK, *B. subtilis*), GLPQ_TREPA (GlpQ, *T. pallidum*), GLPQ_HAEIN (GlpQ, *H. influenzae*), GLPQ_ECOLI (GlpQ, *E. coli*), and UGPQ_ECOLI (UgpQ, *E. coli*).
(TIF)

Figure S2 **Scanning electron microscopy analyses of** ***M. pneumoniae*** **.** Morphology and cell division of *M. pneumoniae* wild type (wt), *glpQ*::Tn, and *mpn566*::Tn mutant strains were compared with each other. All pictures are shown at the same magnification. Scale bar, 1.0 µm.
(TIF)

Figure S3 **Transcription analysis of interesting genes with protein amount changes in the** ***glpQ*** **mutant.** Slot blots were performed with whole RNA extracts of *M. pneumoniae* wild type (wt), *glpQ*::Tn, *mpn566*::Tn, and *glpD*::Tn (control) mutant strains grown in modified Hayflick medium containing either glucose or glycerol as sole carbon source (1% [wt/vol]). A dilution series of RNA extracts was blotted onto a positively charged nylon membrane and probed with a DIG-labeled riboprobe specific for an internal part of a particular open reading frame. Names of riboprobes are given next to each blot. Signals obtained with 1 µg of RNA are shown. Yeast tRNA and *M. pneumoniae* chromosomal DNA served as controls. Genes which had a significant higher protein amount in the *glpQ*::Tn mutant with glucose as carbon source are shown in (A) and genes with significant lower protein amounts in (B). For detailed information on changes of transcript levels see Table S3 and S4.
(TIF)

Table S1 **Proteins with differential expression pattern.** List of proteins that are only expressed in the *M. pneumoniae* wild type strain when grown in modified Hayflick medium containing either glucose or glycerol as sole carbon source (1% [wt/vol]).
(DOC)

Table S2 **Proteins not detected in proteome analysis.** List of proteins that could not be detected in the MS analysis in all tested *M. pneumoniae* strains.
(DOC)

Table S3 **Summary of proteome and transcript analysis in the** ***glpQ*** **mutant GPM81 in the presence of glucose.** Detailed list of significant differences on proteome and transcriptome level in the *glpQ* mutant GPM81 grown with glucose as sole carbon source (1% [wt/vol]).
(DOC)

Table S4 **Summary of proteome and transcript analysis in the glpQ mutant GPM81 in the presence of glycerol.** Detailed list of significant differences on proteome and transcriptome level in the *glpQ* mutant GPM81 grown with glycerol as sole carbon source (1% [wt/vol]).
(DOC)

Table S5 **Oligonucleotides used in this study.** List of oligonucleotides used in this study.
(DOC)

Video S1 **Analysis of the gliding velocity and motility pattern of the** ***M. pneumoniae*** **wild type strain.** Examination of the gliding velocity and motility pattern of the *M. pneumoniae* wild type strain over a 2 min period. Tracks from 6 individual cells are highlighted for better observation.
(MP4)

Video S2 **Analysis of the gliding velocity and motility pattern of the** ***M. pneumoniae glpQ*** **mutant GPM81.** Examination of the gliding velocity and motility pattern of the *M. pneumoniae glpQ* mutant GPM81 over a 2 min period. Tracks from 5 individual cells are highlighted for better observation.
(MP4)

Video S3 **Analysis of the gliding velocity and motility pattern of the** ***M. pneumoniae mpn566*** **mutant GPM82.** Examination of the gliding velocity and motility pattern of the *M. pneumoniae mpn566* mutant GPM82 over a 2 min period. Tracks from 3 individual cells are highlighted for better observation.
(MP4)

## References

[1] Sonenshein AL (2007). Control of key metabolic intersections in *Bacillus subtilis*.. Nat Rev Microbiol.

[2] Görke B, Stülke J (2008). Carbon catabolite repression in bacteria: Many ways to make the most out of nutrients.. Nat Rev Microbiol.

[3] Eisenreich W, Dandekar T, Heesemann J, Goebel W (2010). Carbon metabolism of intracellular bacterial pathogens and possible links to virulence.. Nat Rev Microbiol.

[4] Waites KB, Talkington DF (2004). *Mycoplasma pneumoniae* and its role as human pathogen.. Clin Microbiol Rev.

[5] Tsiodras S, Kelesidis I, Kelesidis T, Stamboulis E, Giamarellou H (2005). Central nervous system manifestations of *Mycoplasma pneumoniae* infection.. J Infect.

[6] Narita M (2009). Pathogenesis of neurogenic manifestations of *Mycoplasma pneumoniae* infection.. Pediatr Neurol.

[7] Narita M (2010). Pathogenesis of extrapulmonary manifestations of *Mycoplasma pneumoniae* infection with special reference to pneumonia.. J Infect Chemother.

[8] Halbedel S, Hames C, Stülke J (2007). Regulation of carbon metabolism in the mollicutes and its relation to virulence.. J Mol Microbiol Biotechnol.

[9] Veldhuizen R, Nag K, Orgeig S, Possmayer F (1998). The role of lipids in pulmonary surfactant.. Biochim Biophys Acta.

[10] Hames C, Halbedel S, Hoppert M, Frey J, Stülke J (2009). Glycerol metabolism is important for cytotoxicity of *Mycoplasma pneumoniae*.. J Bacteriol.

[11] Somerson NL, Walls BE, Chanock RM (1965). Hemolysin of *Mycoplasma pneumoniae*: Tentative identification as a peroxide.. Science.

[12] Pilo P, Vilei EM, Peterhans E, Bonvin-Klotz L, Stoffel MH (2005). A metabolic enzyme as a primary virulence factor of *Mycoplasma mycoides* subsp. *mycoides* small colony.. J Bacteriol.

[13] Bischof DF, Vilei EM, Frey J (2009). Functional and antigenic properties of GlpO from *Mycoplasma mycoides* subsp. *mycoides* SC: Characterization of a flavine adenine dinucleotide-binding site deletion mutant.. Vet Res.

[14] Ohshima N, Yamashita S, Takahashi N, Kuroishi C, Shiro Y (2008). *Escherichia coli* cytosolic glycerophosphodiester phosphodiesterase (UgpQ) requires Mg^2+^, Co^2+^, or Mn^2+^ for its enzyme activity.. J Bacteriol.

[15] Wong KK, Kwan HS (1992). Transcription of *glpT* of *Escherichia coli* K12 is regulated by anaerobiosis and Fnr.. FEMS Microbiol Lett.

[16] Nilsson RP, Beijer L, Rutberg B (1994). The *glpT* and *glpQ* genes of the glycerol regulon in *Bacillus subtilis*.. Microbiology.

[17] Antelmann H, Scharf C, Hecker M (2000). Phosphate starvation-inducible proteins of *Bacillus subtilis*: Proteomics and transcriptional analysis.. J Bacteriol.

[18] Blencke HM, Homuth G, Ludwig H, Mäder U, Hecker M (2003). Transcriptional profiling of gene expression in response to glucose in *Bacillus subtilis*: Regulation of the central metabolic pathways.. Metab Eng.

[19] Fan X, Goldfine H, Lysenko E, Weiser JN (2001). The transfer of choline from the host to the bacterial cell surface requires *glpQ* in *Haemophilus influenzae*.. Mol Microbiol.

[20] Forsgren A, Riesbeck K, Janson H (2008). Protein D of *Haemophilus influenzae*: A protective nontypeable *H. influenzae* antigen and a carrier for pneumococcal conjugate vaccines.. Clin Infect Dis.

[21] Schwan TG, Battisti JM, Porcella SF, Raffel SJ, Schrumpf ME (2003). Glycerol-3-phosphate acquisition in spirochaetes: Distribution and biological activity of glycerophosphodiester phosphodiesterase (GlpQ) among *Borrelia* species.. J Bacteriol.

[22] Commichau FM, Stülke J (2008). Trigger enzymes: Bifunctional proteins active in metabolism and in controlling gene expression.. Mol Microbiol.

[23] Beinert H, Kennedy MC, Stout CD (1996). Aconitase as iron-sulfur protein, enzyme, and iron-regulatory protein.. Chem Rev.

[24] Zhu W, Becker DF (2003). Flavin redox state triggers conformational changes in the PutA protein from *Escherichia coli*.. Biochemistry.

[25] Schmalisch MH, Bachem S, Stülke J (2003). Control of the *Bacillus subtilis* antiterminator protein GlcT by phosphorylation. Elucidation of the phosphorylation chain leading to inactivation of GlcT.. J Biol Chem.

[26] Commichau FM, Herzberg C, Tripal P, Valerius O, Stülke J (2007). A regulatory protein-protein interaction governs glutamate biosynthesis in *Bacillus subtilis*: The glutamate dehydrogenase RocG moonlights in controlling the transcription factor GltC.. Mol Microbiol.

[27] Halbedel S, Stülke J (2007). Tools for the genetic analysis of *Mycoplasma*.. Int J Med Microbiol.

[28] Halbedel S, Busse J, Schmidl SR, Stülke J (2006). Regulatory protein phosphorylation in *Mycoplasma pneumoniae*: A PP2C-type phosphatase serves to dephosphorylate HPr(Ser-P).. J Biol Chem.

[29] Halbedel S, Hames C, Stülke J (2004). *In vivo* activity of enzymatic and regulatory components of the phosphoenolpyruvate:sugar phosphotransferase system in *Mycoplasma pneumoniae.*. J Bacteriol.

[30] Halbedel S, Eilers H, Jonas B, Busse J, Hecker M (2007). Transcription in *Mycoplasma pneumoniae*: Analysis of the promoters of the *ackA* and *ldh* genes.. J Mol Biol.

[31] Schmidl SR, Gronau K, Hames C, Busse J, Becher D (2010). The stability of cytadherence proteins in *Mycoplasma pneumoniae* requires activity of the protein kinase PrkC.. Infect Immun.

[32] Frith MC, Saunders NF, Kobe B, Bailey TL (2008). Discovering sequence motifs with arbitrary insertions and deletions.. PLoS Comput Biol.

[33] Kannan TR, Baseman JB (2006). ADP-ribosylating and vacuolating cytotoxin of *Mycoplasma pneumoniae* represents unique virulence determinant among bacterial pathogens.. Proc Natl Acad Sci U S A.

[34] Vilei EM, Frey J (2001). Genetic and biochemical characterization of glycerol uptake in *Mycoplasma mycoides* subsp. *mycoides* SC: Its impact on H_2_O_2_ production and virulence.. Clin Diagn Lab Immunol.

[35] Yus E, Maier T, Michalodimitrakis K, van Noort V, Yamada T (2009). Impact of genome reduction on bacterial metabolism and its regulation.. Science.

[36] Brown DR, Farley JM, Zacher LA, Carlton JM, Clippinger TL (2001). *Mycoplasma alligatoris* sp. nov., from American alligators.. Int J Syst Evol Microbiol.

[37] Shevchenko DV, Akins DR, Robinson EJ, Li M, Shevchenko OV (1997). Identification of homologs for thioredoxin, peptidyl prolyl *cis-trans* isomerase, glcerophosphodiester phosphodiesterase in outer membrane fractions of *Treponema pallidum*, the syphilis spirochaete.. Infect Immun.

[38] Stebeck CE, Shaffer JM, Arroll TW, Lukehart SA, van Voohis WC (1997). Identification of the *Treponema pallidum* subsp. *pallidum* glcerophosphodiester phosphodiesterase homologue.. FEMS Microbiol Lett.

[39] Jeffery CJ (1999). Moonlighting proteins.. Trends Biochem Sci.

[40] Okazaki Y, Ohshima N, Yoshizawa I, Kamei Y, Marrigio S (2010). A novel glycerophosphodiester phosphodiesterase, GDE5, controls skeletal muscle development via a non-enzymatic mechanism.. J Biol Chem.

[41] Güell M, van Noort V, Yus E, Chen WH, Leigh-Bell J (2009). Transcriptome complexity in a genome-reduced bacterium.. Science.

[42] Schmidl SR, Gronau K, Pietack N, Hecker M, Becher D (2010). The phosphoproteome of the minimal bacterium *Mycoplasma pneumoniae*: Analysis of the complete known Ser/Thr kinome suggests the existence of novel kinases.. Mol Cell Proteomics.

[43] Waldo RH, Popham PL, Romero-Arroyo CE, Mothershed EA, Lee KK (1998). Transcriptional analysis of the *hmw* gene cluster of *Mycoplasma pneumonia*.. J Bacteriol.

[44] Sambrook J, Fritsch EF, Maniatis T (1989). Molecular cloning: A laboratory manual, 2nd ed..

[45] Pich OQ, Burgos R, Planell R, Querol E, Piñol J (2006). Comparative analysis of antibiotic resistance gene markers in *Mycoplasma genitalium*: Application to studies of the minimal gene complement.. Microbiology.

[46] Merzbacher M, Detsch C, Hillen W, Stülke J (2004). *Mycoplasma pneumoniae* HPr kinase/phosphorylase: Assigning functional roles to the P-loop and the HPrK/P signature sequence motif.. Eur J Biochem.

[47] Hames C, Halbedel S, Stülke J (2005). Multiple-mutation reaction: A method for simultaneous introduction of multiple mutations into the *glpK* gene of *Mycoplasma pneumoniae*.. Appl Environ Microbiol.

[48] Larson TJ, Ehrmann M, Boos W (1983). Periplasmic glycerophosphodiester phosphodiesterase of *Escherichia coli*, a new enzyme of the *glp* regulon.. J Biol Chem.

[49] Abramoff MD, Magelhaes PJ, Ram SJ (2004). Image processing with ImageJ.. Biophotonics Int.

[50] Crooks GE, Hon G, Chandonia JM, Brenner SE (2004). WebLogo: A sequence logo generator.. Genome Res.

