# Supplementary figures and images for: A Trigger Enzyme in Mycoplasma pneumoniae: Impact of the Glycerophosphodiesterase GlpQ on Virulence and Gene Expression
Source: PLoS Pathog. 2011 Sep 22;7(9):e1002263. doi: 10.1371/journal.ppat.1002263 (PMC3178575; doi:10.1371/journal.ppat.1002263)

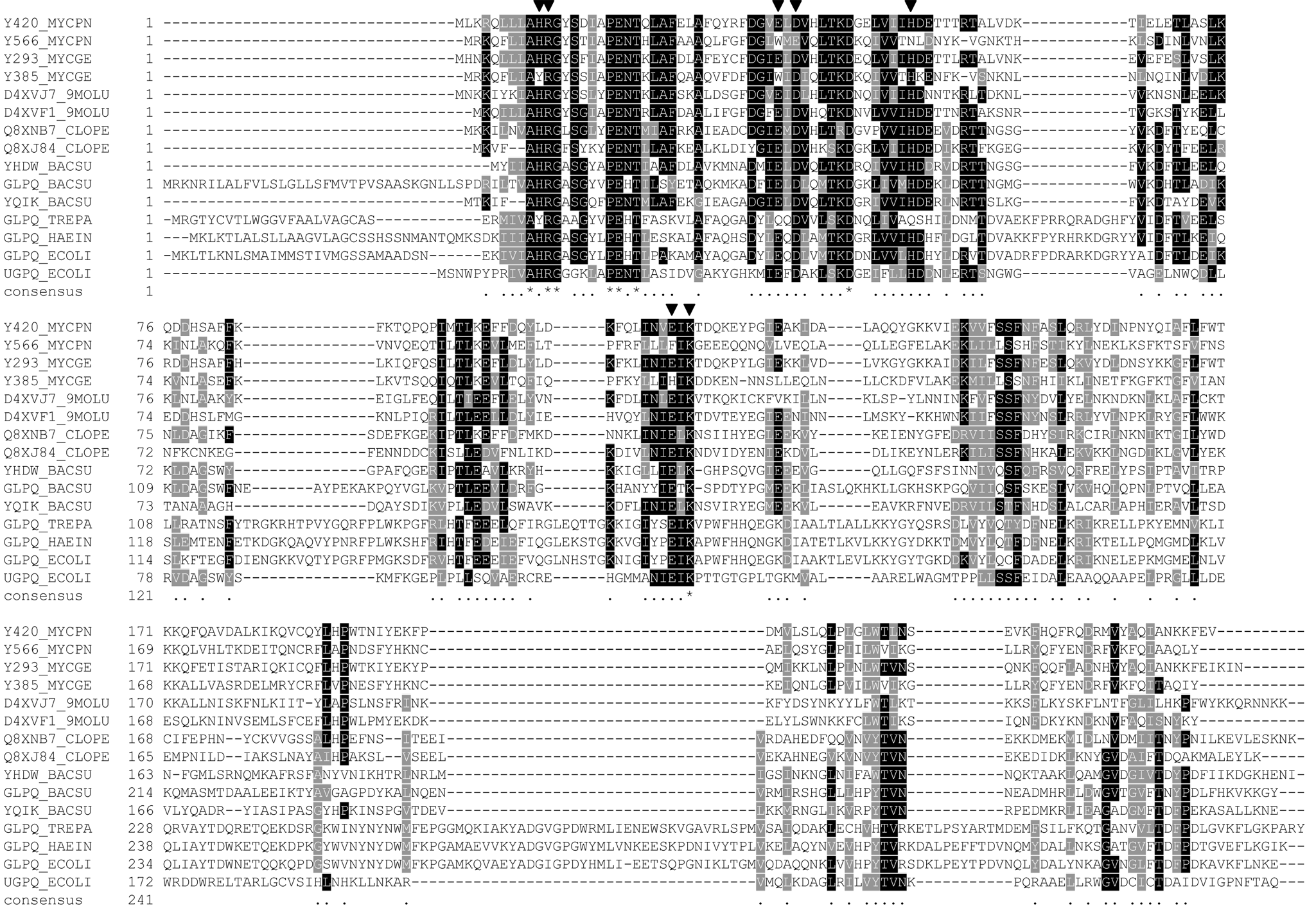

Supplement: Figure S1 — Multiple sequence alignment of GlpQ and MPN566 from M. pneumoniae with orthologous glycerophosphodiesterases of other bacteria. The multiple sequence alignment was performed using ClustalW (http://www.ch.embnet.org/software/ClustalW.html) and represented with BOXSHADE v3.21 (http://www.ch.embnet.org/software/BOX_form.html). Black shading indicates ≥80% identity and grey shading stands for ≥80% similarity. Amino acids that constitute to the strictly conserved active site structure are depicted by black arrows. The UniProtKB entry names of the aligned sequences are Y420_MYCPN (GlpQ, M. pneumoniae), Y566_MYCPN (MPN566, M. pneumoniae), Y293_MYCGE (MG_293, M. genitalium), Y385_MYCGE (MG_385, M. genitalium), D4XVJ7_9MOLU (MALL_0582, M. alligatoris), D4XVF1_9MOLU (MALL_0631, M. alligatoris), Q8XNB7_CLOPE (GlpQ, Clostridium perfringens), Q8XJ84_CLOPE (GlpQ, C. perfringens), YHDW_BACSU (YhdW, B. subtilis), GLPQ_BACSU (GlpQ, B. subtilis), YQIK_BACSU (YqiK, B. subtilis), GLPQ_TREPA (GlpQ, T. pallidum), GLPQ_HAEIN (GlpQ, H. influenzae), GLPQ_ECOLI (GlpQ, E. coli), and UGPQ_ECOLI (UgpQ, E. coli). (TIF) [file ppat.1002263.s001.tif]

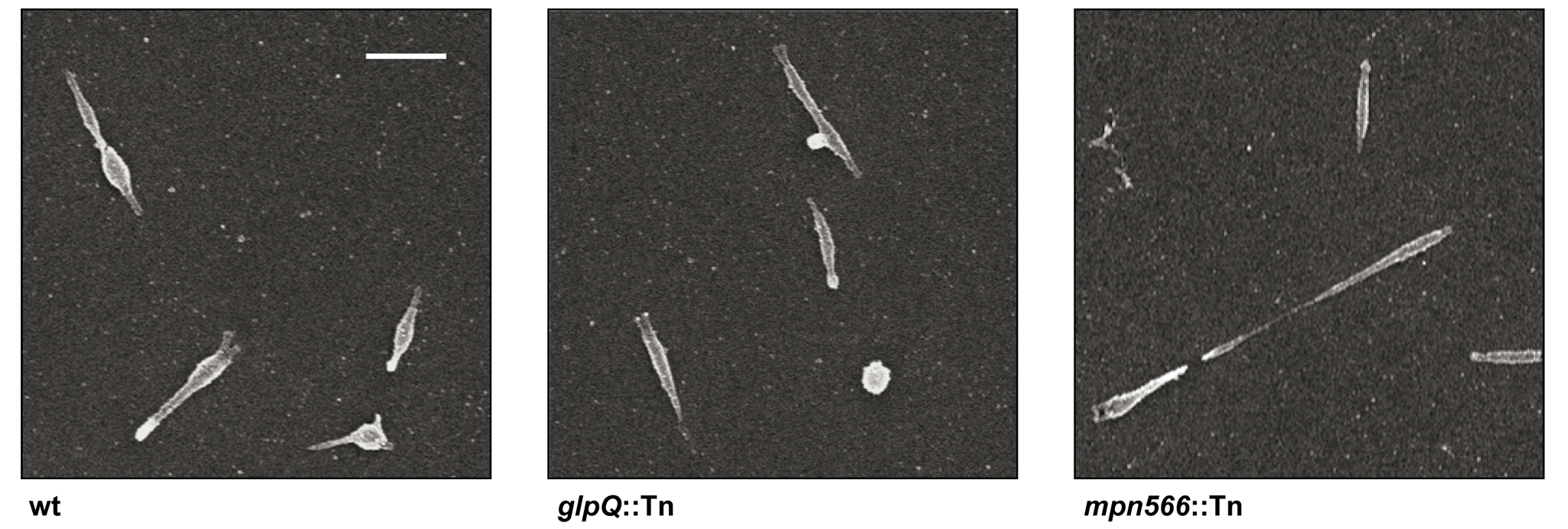

Supplement: Figure S2 — Scanning electron microscopy analyses of M. pneumoniae . Morphology and cell division of M. pneumoniae wild type (wt), glpQ::Tn, and mpn566::Tn mutant strains were compared with each other. All pictures are shown at the same magnification. Scale bar, 1.0 µm. (TIF) [file ppat.1002263.s002.tif]

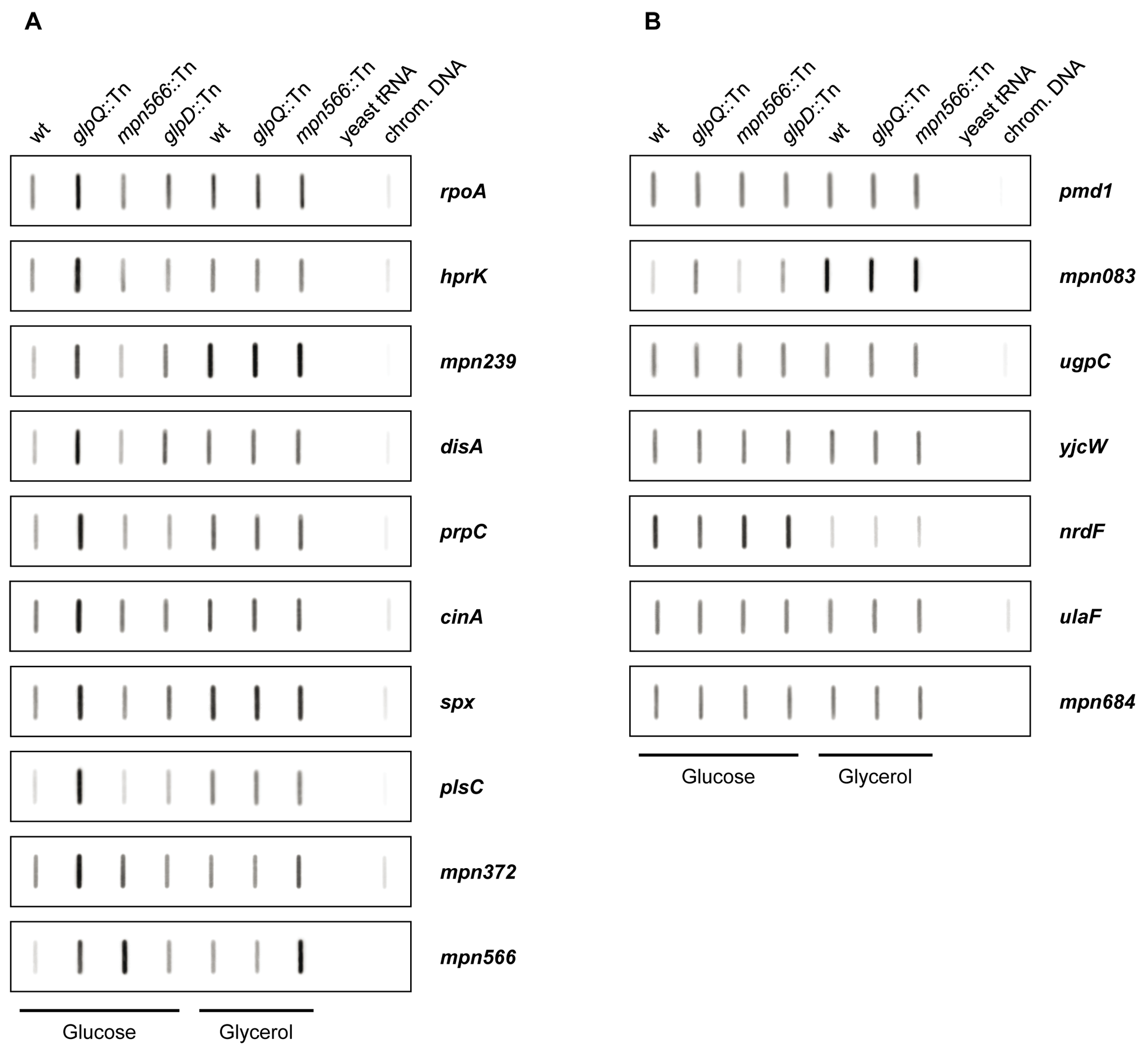

Supplement: Figure S3 — Transcription analysis of interesting genes with protein amount changes in the glpQ mutant. Slot blots were performed with whole RNA extracts of M. pneumoniae wild type (wt), glpQ::Tn, mpn566::Tn, and glpD::Tn (control) mutant strains grown in modified Hayflick medium containing either glucose or glycerol as sole carbon source (1% [wt/vol]). A dilution series of RNA extracts was blotted onto a positively charged nylon membrane and probed with a DIG-labeled riboprobe specific for an internal part of a particular open reading frame. Names of riboprobes are given next to each blot. Signals obtained with 1 µg of RNA are shown. Yeast tRNA and M. pneumoniae chromosomal DNA served as controls. Genes which had a significant higher protein amount in the glpQ::Tn mutant with glucose as carbon source are shown in (A) and genes with significant lower protein amounts in (B). For detailed information on changes of transcript levels see Table S3 and S4. (TIF) [file ppat.1002263.s003.tif]
