# Supplementary material for: A Trigger Enzyme in Mycoplasma pneumoniae: Impact of the Glycerophosphodiesterase GlpQ on Virulence and Gene Expression
Source: PLoS Pathog. 2011 Sep 22;7(9):e1002263. doi: 10.1371/journal.ppat.1002263 (PMC3178575; doi:10.1371/journal.ppat.1002263)
Supplement: Table S1 — Proteins with differential expression pattern. List of proteins that are only expressed in the M. pneumoniae wild type strain when grown in modified Hayflick medium containing either glucose or glycerol as sole carbon source (1% [wt/vol]). (DOC) [file ppat.1002263.s004.doc]

**Table S1. Proteins with differential expression pattern.**

List of proteins that are only expressed in the *M. pneumoniae* wild type strain when grown in modified Hayflick medium containing either glucose or glycerol as sole carbon source (1% wt/vol).

| **Locus**  **name** | **Protein**  **name** | **UniProtKB accession number** | **Locus**  **name** | **Protein**  **name** | **UniProtKB accession number** | **Locus**  **name** | **Protein**  **name** | **UniProtKB accession number** |
| --- | --- | --- | --- | --- | --- | --- | --- | --- |
| **Only identified in the presence of glucose** | | |  |  |  |  |  |  |
| MPN012 | - | P75101 | MPN293 | LspA | P75484 | MPN502 | - | P75285 |
| MPN030 | - | P75084 | MPN333 | - | P75445 | MPN504 | - | P75282 |
| MPN035 | - | P75079 | MPN339 | - | P75439 | MPN505 | - | P75281 |
| MPN068 | SecE | P75048 | MPN345 | HsdR | P75433 | MPN507 | - | P75279 |
| MPN074 | SmpB | P75043 | MPN350 | PlsY | P75428 | MPN512 | - | P75274 |
| MPN095 | - | P75597 | MPN351 | - | P75427 | MPN537 | MucB | P75241 |
| MPN096 | - | P75596 | MPN385 | - | P75397 | MPN564 | Adh | P75214 |
| MPN100 | - | P75592 | MPN404 | - | P75380 | MPN569 | - | P75209 |
| MPN116 | RpmI | P75447 | MPN406 | - | P75378 | MPN575 | - | P75204 |
| MPN130 | - | P75345 | MPN411 | - | P75373 | MPN582 | - | P75198 |
| MPN136 | UgpE | P75262 | MPN414 | - | P75372 | MPN588 | - | Q50339 |
| MPN137 | - | P75261 | MPN431 | - | P75357 | MPN589 | - | Q50338 |
| MPN138 | - | P75260 | MPN435 | - | P75343 | MPN603 | AtpE | Q59550 |
| MPN145 | - | P75141 | MPN440 | - | P75338 | MPN605 | - | Q50325 |
| MPN146 | - | P75140 | MPN450 | - | Q50362 | MPN615 | HsdS | P75180 |
| MPN151 | - | P75035 | MPN455 | CtaD | P75328 | MPN624 | RpmB | P75171 |
| MPN152 | - | P75034 | MPN460 | KtrB | P75323 | MPN651 | MtlA | P75146 |
| MPN163 | - | P75582 | MPN471 | RpmG | P78015 | MPN657 | - | P75134 |
| MPN178 | RpsN | Q50305 | MPN482 | - | Q9EXD7 | MPN659 | TrmD | P75132 |
| MPN214 | - | P75555 | MPN494 | UlaC | P75292 | MPN675 | - | P75117 |
| MPN222 | TilS | P75549 | MPN495 | UlaB | Q9EXD8 | MPN682 | RpmH | P78006 |
| MPN253 | PgsA | P75520 | MPN496 | UlaA | P75291 |  |  |  |
| **Only identified in the presence of glycerol** | | |  |  |  |  |  |  |
| MPN057 | PotC | P75057 | MPN365 | - | P75416 | MPN488 | - | P75297 |
| MPN114 | Cpt2 | P75448 | MPN417 | P69 | P75369 | MPN640 | - | P75157 |
