# Supplementary material for: A Trigger Enzyme in Mycoplasma pneumoniae: Impact of the Glycerophosphodiesterase GlpQ on Virulence and Gene Expression
Source: PLoS Pathog. 2011 Sep 22;7(9):e1002263. doi: 10.1371/journal.ppat.1002263 (PMC3178575; doi:10.1371/journal.ppat.1002263)
Supplement: Table S2 — Proteins not detected in proteome analysis. List of proteins that could not be detected in the MS analysis in all tested M. pneumoniae strains. (DOC) [file ppat.1002263.s005.doc]

**Table S2. Proteins not detected in proteome analysis.**

List of proteins that could not be detected in the MS analysis in all tested *M. pneumoniae* strains.

| **Locus**  **name** | **Protein**  **name** | **UniProtKB accession number** | **Locus**  **name** | **Protein**  **name** | **UniProtKB accession number** | **Locus**  **name** | **Protein**  **name** | **UniProtKB accession number** |
| --- | --- | --- | --- | --- | --- | --- | --- | --- |
| MPN010 | - | P75103 | MPN206 | - | P75570 | MPN466 | - | P75317 |
| MPN014 | DnaE | P75099 | MPN212 | - | P75557 | MPN467 | - | P75316 |
| MPN037 | - | P75077 | MPN242 | SecG | Q9EXD0 | MPN468 | - | P75315 |
| MPN038 | - | P75076 | MPN249 | EngC | P75523 | MPN485 | - | P75300 |
| MPN039 | - | P75075 | MPN270 | - | Q9EXD1 | MPN486 | - | P75299 |
| MPN040 | - | P75074 | MPN274 | - | P75503 | MPN497 | - | P75290 |
| MPN041 | - | P75073 | MPN282 | - | P75495 | MPN500 | - | P75287 |
| MPN042 | - | P75072 | MPN283 | - | P75494 | MPN503 | - | P75283 |
| MPN048 | - | P75066 | MPN285 | PrrB | P75492 | MPN508 | - | P75278 |
| MPN049 | - | P75065 | MPN286 | - | P75491 | MPN510 | - | P75276 |
| MPN054 | - | P75060 | MPN289 | HsdS1B | P75488 | MPN511 | - | P75275 |
| MPN056 | PotB | P75058 | MPN290 | - | P75487 | MPN513 | - | P75273 |
| MPN069 | RpmG2 | P56850 | MPN304 | ArcA | P75475 | MPN514 | - | P75272 |
| MPN085 | - | P75608 | MPN305 | ArcA | P75475 | MPN525 | - | P75253 |
| MPN086 | - | P75607 | MPN306 | ArcB | P75473 | MPN527 | - | P75251 |
| MPN087 | - | P75606 | MPN313 | - | P75468 | MPN534 | - | P75244 |
| MPN088 | - | P75605 | MPN334 | BcrA | P75444 | MPN535 | RuvA | P75243 |
| MPN089 | HsdS | P75604 | MPN335 | - | P75443 | MPN536 | RuvB | P75242 |
| MPN091 | - | P75602 | MPN343 | - | P75435 | MPN540 | RpmF | P75238 |
| MPN092 | - | P75600 | MPN346 | - | P75432 | MPN565 | - | P75213 |
| MPN093 | - | P75599 | MPN347 | HsdR | P75431 | MPN570 | - | P75208 |
| MPN097 | - | P75595 | MPN363 | - | P75418 | MPN571 | LcnDR3 | P75207 |
| MPN098 | - | P75594 | MPN364 | - | P75417 | MPN577 | - | P75203 |
| MPN099 | - | P75593 | MPN366 | - | P75415 | MPN578 | - | P75202 |
| MPN101 | - | P75568 | MPN367 | - | P75414 | MPN579 | - | P75201 |
| MPN102 | - | P75567 | MPN369 | - | P75412 | MPN580 | - | P75200 |
| MPN103 | - | P75566 | MPN370 | - | P75411 | MPN581 | - | P75199 |
| MPN107 | - | P75562 | MPN371 | - | P75410 | MPN583 | - | P75197 |
| MPN108 | - | P75561 | MPN373 | - | P75408 | MPN584 | - | P75196 |
| MPN110 | - | P75452 | MPN374 | - | P75407 | MPN586 | - | P75194 |
| MPN111 | - | P75451 | MPN375 | - | P75406 | MPN587 | - | P75193 |
| MPN112 | - | P75450 | MPN388 | - | Q9EXD4 | MPN590 | - | Q50337 |
| MPN113 | - | P75449 | MPN403 | - | P75381 | MPN593 | - | Q50334 |
| MPN127 | - | P75348 | MPN405 | - | P75379 | MPN594 | - | P75191 |
| MPN128 | - | P75347 | MPN409 | - | P75375 | MPN612 | - | P75183 |
| MPN129 | - | P75346 | MPN412 | - | Q9EXD5 | MPN613 | - | P75182 |
| MPN131 | - | P75267 | MPN413 | - | Q9EXD6 | MPN614 | - | P75181 |
| MPN132 | - | P75266 | MPN437 | - | P75341 | MPN626 | - | P75169 |
| MPN143 | - | P75143 | MPN438 | - | P75340 | MPN633 | - | P75164 |
| MPN144 | - | P75142 | MPN439 | - | P75339 | MPN634 | - | P75163 |
| MPN147 | - | P75139 | MPN441 | - | P75337 | MPN635 | - | P75162 |
| MPN149 | - | P75037 | MPN442 | - | P75336 | MPN637 | CdsA | P75160 |
| MPN150 | - | P75036 | MPN448 | - | Q50364 | MPN644 | - | P75153 |
| MPN160 | - | P75585 | MPN451 | Come3 | Q50361 | MPN648 | - | P75149 |
| MPN188 | RpmJ | P52864 | MPN457 | - | P75326 | MPN649 | - | P75148 |
| MPN201 | - | Q50287 | MPN458 | - | P75325 | MPN650 | - | P75147 |
| MPN202 | - | Q50286 | MPN462 | - | P75321 | MPN654 | - | P75137 |
| MPN203 | - | Q50284 | MPN463 | - | P75320 | MPN666 | - | P75125 |
| MPN204 | - | P75572 | MPN464 | - | P75319 | MPN676 | - | P75116 |
| MPN205 | - | P75571 | MPN465 | - | P75318 | MPN681 | RnpA | P75111 |
