# Supplementary material for: A Trigger Enzyme in Mycoplasma pneumoniae: Impact of the Glycerophosphodiesterase GlpQ on Virulence and Gene Expression
Source: PLoS Pathog. 2011 Sep 22;7(9):e1002263. doi: 10.1371/journal.ppat.1002263 (PMC3178575; doi:10.1371/journal.ppat.1002263)
Supplement: Table S3 — Summary of proteome and transcript analysis in the glpQ mutant GPM81 in the presence of glucose. Detailed list of significant differences on proteome and transcriptome level in the glpQ mutant GPM81 grown with glucose as sole carbon source (1% [wt/vol]). (DOC) [file ppat.1002263.s006.doc]

**Table S3. Summary of proteome and transcript analysis in the *glpQ* mutant GPM81 in the presence of glucose.**

Detailed list of significant differences on proteome and transcriptome level in the *glpQ* mutant GPM81 grown with glucose as sole carbon source (1% wt/vol).

| **Locus name** | **Protein name** | **UniProtKB accession number** | **Protein function** | **COG^a^** | **Molecular weight**  **(kDa)** | **Isoelectric point**  **(pI)** | **Fold-change^b^** | |
| --- | --- | --- | --- | --- | --- | --- | --- | --- |
|  |  |  |  |  |  |  | **Protein**  **level** | **Transcript**  **level** |
| **Induced** | |  |  |  |  |  |  |  |
| MPN023 | MetG | P75091 | Methionyl-tRNA synthetase | J | 59.26 | 6.63 | 2.25 ± 0.22 | na |
| MPN043 | GlpF | P75071 | Glycerol uptake facilitator | G | 28.31 | 9.33 | 10.79 ± 1.06 | 5.69 ± 0.36 |
| MPN060 | MetK | P78003 | *S*-adenosylmethionine synthetase | J | 42.56 | 6.07 | 2.33 ± 0.11 | na |
| MPN162 | - | P75583 | Uncharacterized lipoprotein | S | 36.10 | 6.19 | 9.96 ± 0.97 | 5.23 ± 0.23 |
| MPN179 | RpsH | Q50304 | 30S ribosomal protein S8 | J | 15.88 | 10.32 | 2.63 ± 0.33 | na |
| MPN191 | RpoA | Q50295 | RNA polymerase subunit α | K | 36.66 | 6.98 | 2.30 ± 0.04 | 2.18 ± 0.17 |
| MPN209 | PacL | P78036 | Cation-transporting P-type ATPase | P | 94.97 | 6.38 | 2.49 ± 0.26 | na |
| MPN221 | Pth | P78034 | Peptidyl-tRNA hydrolase | J | 21.42 | 9.52 | 2.43 ± 0.14 | na |
| MPN223 | HPrK | P75548 | HPr kinase phosphorylase | O | 35.24 | 8.89 | 2.90 ± 0.29 | 2.83 ± 0.09 |
| MPN239 | - | P75532 | HTH-type transcriptional regulator | K | 25.85 | 9.50 | 2.53 ± 0.17 | 3.16 ± 0.14 |
| MPN244 | DisA | P75528 | Uncharacterized protein | L | 22.73 | 9.38 | 2.72 ± 0.30 | 3.39 ± 0.19 |
| MPN247 | PrpC | P75525 | Protein phosphatase | O | 29.69 | 8.57 | 2.43 ± 0.18 | 2.91 ± 0.12 |
| MPN248 | PrkC | P75524 | Serine threonine-protein kinase | O | 44.88 | 9.08 | 2.33 ± 0.11 | na |
| MPN254 | CinA | Q9EXC9 | Uncharacterized protein | S | 16.96 | 8.81 | 2.60 ± 0.27 | 2.11 ± 0.10 |
| MPN266 | Spx | P75509 | Transcriptional regulator | K | 16.81 | 9.81 | 2.71 ± 0.13 | 2.23 ± 0.07 |
| MPN279 | LepA | P75498 | GTP-binding protein | J | 66.16 | 7.58 | 2.34 ± 0.18 | na |
| MPN299 | PlsC | P75479 | 1-acyl-*sn*-glycerol 3-phosphate acyltransferase | I | 30.42 | 9.79 | 3.20 ± 0.35 | 6.43 ± 0.31 |
| MPN300 | ScpA | P75478 | Segregation and condensation protein A | U | 59.52 | 8.15 | 2.45 ± 0.18 | na |
| MPN338 | - | P75440 | Uncharacterized protein | S | 74.34 | 5.60 | 2.51 ± 0.23 | na |
| MPN340 | PcrA | P75438 | DNA helicase II | L | 60.53 | 6.23 | 2.43 ± 0.21 | na |
| MPN357 | LigA | P78021 | DNA ligase | L | 73.97 | 8.40 | 2.58 ± 0.30 | na |
| MPN359 | - | P75421 | Uncharacterized protein | S | 30.56 | 9.67 | 2.34 ± 0.24 | na |
| MPN372 | - | P75409 | ADP-ribosylating toxin CARDS | V | 68.06 | 5.63 | 2.91 ± 0.21 | 2.72 ± 0.20 |
| MPN407 | - | P75377 | Lipase | I | 101.09 | 5.81 | 2.13 ± 0.11 | na |
| MPN408 | - | P75376 | Uncharacterized lipoprotein | S | 83.35 | 9.16 | 3.57 ± 0.26 | na |
| MPN425 | FtsY | P75362 | Cell division protein homolog | D | 38.78 | 6.85 | 2.34 ± 0.21 | na |
| MPN433 | CbiO | P75355 | Metal ion ABC transporter | P | 30.77 | 8.41 | ns | 7.56 ± 0.39 |
| MPN456 | - | P75327 | Uncharacterized lipoprotein | S | 110.52 | 6.33 | 2.35 ± 0.18 | na |
| MPN518 | - | P75269 | Uncharacterized protein | S | 40.72 | 8.93 | 3.42 ± 0.12 | na |
| MPN544 | - | P75234 | Uncharacterized protein | S | 76.77 | 7.50 | 5.14 ± 0.48 | na |
| MPN552 | - | P75226 | Uncharacterized protein | S | 30.91 | 5.66 | 4.04 ± 0.41 | na |
| MPN566 | - | P75212 | Similar to glycerophosphoryldiester phosphodiesterase | S | 27.72 | 9.24 | 2.89 ± 0.16 | 4.98 ± 0.24 |
| MPN625 | - | P75170 | Osmotical inducible protein C-like protein | O | 15.47 | 6.50 | 2.56 ± 0.13 | na |
| MPN664 | DegV | P75127 | Uncharacterized protein | S | 26.84 | 9.11 | 2.63 ± 0.30 | na |
| **Repressed** | |  |  |  |  |  |  |  |
| MPN015 | - | P75098 | Uncharacterized protein | S | 33.45 | 9.89 | 0.27 ± 0.02 | na |
| MPN018 | Pmd1 | P75095 | ABC transporter ATP-binding protein | S | 68.93 | 9.07 | 0.20 ± 0.05 | sa |
| MPN019 | MsbA | P75094 | ABC transporter ATP-binding protein | S | 71.15 | 9.52 | 0.17 ± 0.04 | na |
| MPN055 | PotA | P75059 | Spermidine putrescine import  ATP-binding protein | E | 65.13 | 9.10 | 0.26 ± 0.02 | na |
| MPN083 | - | P75610 | Uncharacterized lipoprotein | S | 59.97 | 7.69 | nd | 2.81 ± 0.16 |
| MPN084 | - | P75609 | Uncharacterized lipoprotein | S | 59.56 | 5.98 | 0.32 ± 0.03 | na |
| MPN090 | - | P75603 | Adhesin P1 | M | 37.44 | 9.52 | 0.47 ± 0.03 | na |
| MPN134 | UgpC | P75264 | *sn*-glycerol-3-phosphate transport system permease | G | 66.47 | 9.11 | 0.39 ± 0.06 | sa |
| MPN258 | YjcW | P75516 | Sugar ABC transporter ATP-binding protein | S | 64.87 | 9.55 | 0.21 ± 0.04 | sa |
| MPN259 | - | P75515 | Sugar ABC transporter permease | S | 58.59 | 9.71 | 0.22 ± 0.06 | na |
| MPN260 | RbsC | P75514 | Sugar ABC transporter permease | S | 33.49 | 9.42 | 0.37 ± 0.01 | na |
| MPN284 | - | P75493 | Uncharacterized lipoprotein | S | 87.17 | 9.16 | ns | 0.25 ± 0.05 |
| MPN322 | NrdF | P75461 | Ribonucleoside-diphosphate reductase subunit β | F | 39.41 | 5.47 | 0.38 ± 0.05 | ns |
| MPN323 | NrdI | P75460 | Ribonucleoside-diphosphate reductase stimulatory protein | F | 17.15 | 7.79 | 0.29 ± 0.06 | na |
| MPN324 | NrdE | P78027 | Ribonucleoside-diphosphate reductase subunit α | F | 82.38 | 6.63 | 0.40 ± 0.05 | na |
| MPN420 | GlpQ | P75367 | Glycerophosphoryldiester phosphodiesterase | C | 28.37 | 6.32 | nd | na |
| MPN449 | - | Q50363 | Uncharacterized protein | S | 50.55 | 9.32 | 0.10 ± 0.04 | na |
| MPN498 | UlaF | P75289 | L-ribulose-5-phosphate 4-epimerase | C | 27.09 | 6.44 | 0.20 ± 0.04 | sa |
| MPN506 | - | P75280 | Uncharacterized lipoprotein | S | 87.50 | 9.24 | 0.10 ± 0.03 | 0.07 ± 0.05 |
| MPN684 | - | P75109 | ABC transporter permease | S | 209.45 | 8.23 | 0.32 ± 0.03 | sa |
| MPN685 | CysA | Q50316 | ABC transport ATP-binding protein | S | 32.24 | 9.69 | 0.21 ± 0.04 | na |

^a^ Abbreviations: COG, Cluster of orthologous groups of proteins; C, Energy production and conversion; D, Cell cycle control, cell division, and chromosome partitioning; E, Amino acid transport and metabolism; F, Nucleotide transport and metabolism; G, Carbohydrate transport and metabolism; I, Lipid metabolism; J, Translation, ribosomal structure, and biogenesis; K, Transcription; L, Replication, recombination, and repair; M, Cell wall, membrane, and envelope biogenesis; O, Posttranslational modification, protein turnover, and chaperones; P, Inorganic ion transport and metabolism; S, Function unknown; U, Intracellular trafficking, secretion, and vesicular transport; V, Defense mechanisms.

^b^ Fold-change cut off ≥ 2.0 and ≤ 0.5, respectively (*glpQ* mutant strain *vs*. wild type). Abbreviations: na, not available; nd, not detectable; ns, no significant difference; sa, similar amount.
