# Supplementary material for: A Trigger Enzyme in Mycoplasma pneumoniae: Impact of the Glycerophosphodiesterase GlpQ on Virulence and Gene Expression
Source: PLoS Pathog. 2011 Sep 22;7(9):e1002263. doi: 10.1371/journal.ppat.1002263 (PMC3178575; doi:10.1371/journal.ppat.1002263)
Supplement: Table S4 — Summary of proteome and transcript analysis in the glpQ mutant GPM81 in the presence of glycerol. Detailed list of significant differences on proteome and transcriptome level in the glpQ mutant GPM81 grown with glycerol as sole carbon source (1% [wt/vol]). (DOC) [file ppat.1002263.s007.doc]

**Table S4. Summary of proteome and transcript analysis in the *glpQ* mutant GPM81 in the presence of glycerol.**

Detailed list of significant differences on proteome and transcriptome level in the *glpQ* mutant GPM81 grown with glycerol as sole carbon source (1% wt/vol).

| **Locus name** | **Protein name** | **UniProtKB accession number** | **Protein function** | **COG^a^** | **Molecular weight**  **(kDa)** | **Isoelectric point**  **(pI)** | **Fold-change^b^** | |
| --- | --- | --- | --- | --- | --- | --- | --- | --- |
|  |  |  |  |  |  |  | **Protein**  **level** | **Transcript**  **level** |
| **Induced** | |  |  |  |  |  |  |  |
| MPN043 | GlpF | P75071 | Glycerol uptake facilitator | G | 28.31 | 9.33 | 5.83 ± 0.76 | 3.32 ± 0.34 |
| MPN162 | - | P75583 | Uncharacterized lipoprotein | S | 36.10 | 6.19 | 5.41 ± 0.83 | 2.81 ± 0.47 |
| MPN433 | CbiO | P75355 | Metal ion ABC transporter | P | 30.77 | 8.41 | 5.66 ± 0.49 | 2.92 ± 0.24 |
| MPN444 | - | P75334 | Uncharacterized lipoprotein | S | 146.28 | 7.89 | 2.74 ± 0.09 | na |
| MPN489 | - | P75296 | Uncharacterized lipoprotein | S | 143.06 | 9.14 | 2.40 ± 0.20 | na |
| **Repressed** | |  |  |  |  |  |  |  |
| MPN284 | - | P75493 | Uncharacterized lipoprotein | S | 87.17 | 9.16 | 0.36 ± 0.02 | 0.35 ± 0.04 |
| MPN288 | - | P75489 | Uncharacterized lipoprotein | S | 86.89 | 9.07 | 0.29 ± 0.03 | na |
| MPN420 | GlpQ | P75367 | Glycerophosphoryldiester phosphodiesterase | C | 28.37 | 6.32 | nd | na |
| MPN506 | - | P75280 | Uncharacterized lipoprotein | S | 87.50 | 9.24 | 0.18 ± 0.04 | 0.16 ± 0.02 |
| MPN673 | - | P75118 | Uncharacterized protein | S | 19.47 | 9.16 | 0.21 ± 0.03 | na |

^a^ Abbreviations: COG, Cluster of orthologous groups of proteins; C, Energy production and conversion; G, Carbohydrate transport and metabolism; P, Inorganic ion transport and metabolism; S, Function unknown.

^b^ Fold-change cut off ≥ 2.0 and ≤ 0.5, respectively (*glpQ* mutant strain *vs*. wild type). Abbreviations: na, not available; nd, not detectable.
