# Supplementary material for: A Trigger Enzyme in Mycoplasma pneumoniae: Impact of the Glycerophosphodiesterase GlpQ on Virulence and Gene Expression
Source: PLoS Pathog. 2011 Sep 22;7(9):e1002263. doi: 10.1371/journal.ppat.1002263 (PMC3178575; doi:10.1371/journal.ppat.1002263)
Supplement: Table S5 — Oligonucleotides used in this study. List of oligonucleotides used in this study. (DOC) [file ppat.1002263.s008.doc]

**Table S5.** **Oligonucleotides used in this study.**

| **Primer** | **Sequence (5’→3’)^ab^** | **Description** |
| --- | --- | --- |
| CH61 | ATGCCAAATCCTGTTAGATTTGTTTAC | *mpn372* probe fw |
| CH63 | CTAATACGACTCACTATAGGGAGAGCCGGGTGGTGAGCATG | *mpn372* probe rev |
| SH03 | gagtacccggattaaagcggg | *hprK* probe fw |
| SH04 | CTAATACGACTCACTATAGGGAGAcattaactggatttcggtgcgctg | *hprK* probe rev |
| SH05 | CAGGTAACGGTGCTGGTTCG | *glpF* probe fw |
| SH06 | CTAATACGACTCACTATAGGGAGAATTGCAGTACCAGTAGCGGC | *glpF* probe rev |
| SH29 | ATGAGTGAGCTAACTCACAG | Screening primer for transposon insertions |
| SH30 | CAATACGCAAACCGCCTC | Screening primer for transposon insertions |
| SH62 | TAGAATTTTATGGTGGTAGAG | *aac-ahpD* probe fw |
| SH63 | CTAATACGACTCACTATAGGGAGAACACTATCATAACCACTACC | *aac-ahpD* probe rev |
| SH66 | AAAGTCGACATGGACAGCACCAACCAAAAC | *prpC* probe fw (*Sal*I) |
| SH73 | CTAATACGACTCACTATAGGGAGAGACCATCAGAGCACAACAG | *prpC* probe rev |
| SS34 | AAAGAGCTCGATGCTTAAACGACAACTTCTGCTAGC | *glpQ* gene fw (*Sac*I) |
| SS35 | TATAGGATCCTTACACTTCAAACTTCTTGTTGGCAATTTG | *glpQ* gene rev (*Bam*HI) |
| SS36 | P_GCCTTTTTGTTTTGGACGAAAAAGCAGTTCCAAG | *glpQ* A507G |
| SS37 | P_CAGTATCTCCATCCCTGGACAAACATTTACG | *glpQ* A576G |
| SS38 | P_CCTTTAGGGCTGTGGACGCTTAACAGTG | *glpQ* A639G |
| SS39 | AAAGAGCTCGATGCGCAAACAGTTTTTAATTGCACAC | *mpn566* gene fw (*Sac*I) |
| SS40 | TATAGGATCCTTAGTAAAGTTGTGCTGCTATTTGAAATTTAAC | *mpn566* gene rev (*Bam*HI) |
| SS42 | CAACTTCTGCTAGCACACCG | *glpQ* probe fw |
| SS43 | CTAATACGACTCACTATAGGGAGAGCTATTTGGTAGTTGGGGTTAATG | *glpQ* probe rev |
| SS44 | GCAAACAGTTTTTAATTGCACACCG | *mpn566* probe fw |
| SS45 | CTAATACGACTCACTATAGGGAGAGCTCTTTAACTTTTCGTTGAGGTAC | *mpn566* probe rev |
| SS123 | GAATCAGTTTCTCCCTTAGAATATGC | *nrdF* probe fw |
| SS124 | CTAATACGACTCACTATAGGGAGAGTCTTTCCCGGTGTAATAGGG | *nrdF* probe rev |
| SS127 | CCAACAGCGCTTTTATTCTCGG | *mpn083* probe fw |
| SS128 | CTAATACGACTCACTATAGGGAGAGGACATTAGGTTTGGTGTACTTAC | *mpn083* probe rev |
| SS129 | CCTTGTTAGTTGCGCCACAC | *mpn162* probe fw |
| SS130 | CTAATACGACTCACTATAGGGAGACACCTTCGTGGTGATCATGATC | *mpn162* probe rev |
| SS131 | GGCTTAGTCATCCACACTTGG | *ulaF* probe fw |
| SS132 | CTAATACGACTCACTATAGGGAGACCACTGCATCCTTGCCATTC | *ulaF* probe rev |
| SS135 | GGTTTTACCCGTTTTTGTGTTAATGC | *plsC* probe fw |
| SS136 | CTAATACGACTCACTATAGGGAGAGCCCGATTTAAACTCACCAATTTG | *plsC* probe rev |
| SS137 | CCGTTACATTCTCCTTAAAATTCAAAG | *mpn239* probe fw |
| SS138 | CTAATACGACTCACTATAGGGAGAGCTCTACCACAATGCCGTTG | *mpn239* probe rev |
| SS139 | ATGCTTAAGAAAAAAGTTAATAATGATGCTG | *spx* probe fw |
| SS140 | CTAATACGACTCACTATAGGGAGATTACTTCTTTACTGTACGCACTTTAGG | *spx* probe rev |
| SS141 | CCGCTTTTCACCTTTGCACAG | *pmd1* probe fw |
| SS142 | CTAATACGACTCACTATAGGGAGAGAGTTAGTGGCAATAGCAAAGGC | *pmd1* probe rev |
| SS143 | GCTAATGTAGATGTTAACCTCACG | *yjcW* probe fw |
| SS144 | CTAATACGACTCACTATAGGGAGACTCAAAACAGCGGTTGGTTCATC | *yjcW* probe rev |
| SS145 | CCTGATTTATGACAAAGAAGGTAACC | *mpn684* probe fw |
| SS146 | CTAATACGACTCACTATAGGGAGAGGGATTAGTCTCTAACCAAAACTG | *mpn684* probe rev |
| SS147 | GAATATTGTAGTCGACTTTGGTGAG | *ugpC* probe fw |
| SS148 | CTAATACGACTCACTATAGGGAGAGGTTAGCTTCCAGTTCACTCTG | *ugpC* probe rev |
| SS168 | GCTAAATCATAAGCTCACCATTGC | *cinA* probe fw |
| SS169 | CTAATACGACTCACTATAGGGAGAGCTGCATCTTGTTCTCTTAAACC | *cinA* probe rev |
| SS170 | CTAACCCCAACTACGGCATC | *rpoA* probe fw |
| SS171 | CTAATACGACTCACTATAGGGAGACCCCAAAGAATTAATCATTTCACGG | *rpoA* probe rev |
| SS172 | CTTAATCTTAGCGCTTACCTTGTTG | *disA* probe fw |
| SS173 | CTAATACGACTCACTATAGGGAGAGTTTTAATCACGCCGCGCAC | *disA* probe rev |
| SS176 | CTCAATTTTAGCCTCAAACCCAAC | *cbiO* probe fw |
| SS177 | CTAATACGACTCACTATAGGGAGACCGCTTTAGTTTGCTAGCTAGC | *cbiO* probe rev |
| SS190 | GCTGCCTGTGGTACAAAGG | *mpn506* probe fw |
| SS191 | CTAATACGACTCACTATAGGGAGAGTACCTTCTTTCTTTTTAGCGTTGTTC | *mpn506* probe rev |
| SS192 | P_GATGGTTTGGAGATGGATGTGCAACTCAC | *mpn566* T106G/G107A/A114T |
| SS193 | P_CGTTTCGCTTTCTCTTGCTTGAAATTAAGGGCG | *mpn566* T328G/T329A/T330A |
| SS194 | P_GTAGTCACTCATGATGACAACTATAAGGTAGGAAATAAAACC | *mpn566* A151C/T154G/T155A/A156T |
| SS199 | CCAATTAAAGCTCCTTCAAAGAATGG | *mpn284* probe fw |
| SS200 | CTAATACGACTCACTATAGGGAGACGATCGTATTGTTCGTCATTAATTTTG | *mpn284* probe rev |
| SS245 | TTTGAATTCTTACACTTCAAACTTCTTGTTGGCAATTTG | *glpQ* gene rev, complementation (*Eco*RI) |
| SS267 | AAACTCGAGCAAATCTAAATACGGTTTTCCTCTCAC | *glpQ* gene fw, complementation (*Xho*I) |
| SS272 | GGAGCGATTACAGAATTAGGAAGC | *tet* probe fw |
| SS273 | CTAATACGACTCACTATAGGGAGACTCTAAAAGGTCATCGTTTCCCTC | *tet* probe rev |

^a^ Restriction sites and T7-promotors are underlined, respectively.

^b^ The “P” at the 5’ end of primer sequences indicates phosphorylation.
